# recount workflow: Accessing over 70,000 human RNA-seq samples with Bioconductor

**DOI:** 10.12688/f1000research.12223.1

**Published:** 2017-08-24

**Authors:** Leonardo Collado-Torres, Abhinav Nellore, Andrew E. Jaffe

**Affiliations:** 1 Lieber Institute for Brain Development, Baltimore, MD, 21205, USA; 2 Center for Computational Biology, Johns Hopkins University, Baltimore, MD, 21205 , USA; 3 Department of Biomedical Engineering, Oregon Health and Science University, Portland, OR, 97239, USA; 4 Department of Surgery, Oregon Health and Science University, Portland, OR, 97239, USA; 5 Computational Biology Program, Oregon Health and Science University, Portland, OR, 97239, USA; 6 Department of Biostatistics, Johns Hopkins Bloomberg School of Public Health, Baltimore, MD, 21205, USA; 7 Department of Mental Health, Johns Hopkins Bloomberg School of Public Health, Baltimore, MD, 21205, USA

**Keywords:** RNA-seq, visualization, differential expression, human, Bioconductor, genomics, bioinformatics, GTEx, TCGA, SRA

## Abstract

The recount2 resource is composed of over 70,000 uniformly processed human RNA-seq samples spanning TCGA and SRA, including GTEx. The processed data can be accessed via the recount2 website and the ***recount***Bioconductor package. This workflow explains in detail how to use the ***recount***package and how to integrate it with other Bioconductor packages for several analyses that can be carried out with the recount2 resource. In particular, we describe how the coverage count matrices were computed in recount2 as well as different ways of obtaining public metadata, which can facilitate downstream analyses. Step-by-step directions show how to do a gene-level differential expression analysis, visualize base-level genome coverage data, and perform an analyses at multiple feature levels. This workflow thus provides further information to understand the data in recount2 and a compendium of R code to use the data.

## Introduction

RNA sequencing (RNA-seq) is now the most widely used high-throughput assay for measuring gene expression. In a typical RNA-seq experiment, several million reads are sequenced per sample. The reads are often aligned to the reference genome using a splice-aware aligner to identify where reads originated. Resulting alignment files are then used to compute count matrices for several analyses such as identifying differentially expressed genes. The Bioconductor project ^1^ has many contributed packages that specialize in analyzing this type of data and previous workflows have explained how to use them ^2– 4^. Initial steps are typically focused on generating the count matrices. Some pre-computed matrices have been made available via the ReCount project ^5^ or Bioconductor Experiment data packages such as the airway dataset ^6^. The pre-computed count matrices in ReCount have been useful to RNA-seq methods developers and to researchers seeking to avoid the computationally intensive process of creating these matrices. In the years since ReCount was published, hundreds of new RNA-seq projects have been carried out, and researchers have shared the data publicly.

We recently uniformly processed over 70,000 publicly available human RNA-seq samples, and made the data available via the recount2 resource at jhubiostatistics.shinyapps.io/recount/ ^7^. Samples in recount2 are grouped by project (over 2,000) originating from the Sequence Read Archive, the Genotype-Tissue Expression study (GTEx) and the Cancer Genome Atlas (TCGA). The processed data can be accessed via the recount Bioconductor package available at bioconductor.org/packages/recount. Together, recount2 and the recount Bioconductor package should be considered a successor to ReCount.

Due to space constraints, the recount2 publication ^7^ did not cover how to use the recount package and other useful information for carrying out analyses with recount2 data. We describe how the count matrices in recount2 were generated. We also review the R code necessary for using the recount2 data, whose details are important because some of this code involves multiple Bioconductor packages and changing default options. We further show: a) how to augment metadata that comes with datasets with metadata learned from natural language processing of associated papers as well as expression data b) how to perform differential expression analyses, and c) how to visualize the base-pair data available from recount2.

## Analysis of RNA-seq data available at recount2

### recount2 overview

**Figure 1.**
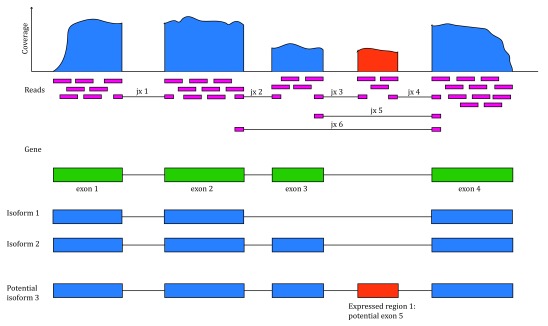
Overview of the data available in recount2. Reads (pink boxes) aligned to the reference genome can be used to compute a base-pair coverage curve and identify exon-exon junctions (split reads). Gene and exon count matrices are generated using annotation information providing the gene (green boxes) and exon (blue boxes) coordinates together with the base-level coverage curve. The reads spanning exon-exon junctions (jx) are used to compute a third count matrix that might include unannotated junctions (jx 3 and 4). Without using annotation information, expressed regions (orange box) can be determined from the base-level coverage curve to then construct data-driven count matrices.

The recount2 resource provides expression data summarized at different feature levels to enable novel cross-study analyses. Generally when investigators use the term *expression*, they think about gene expression. But more information can be extracted from RNA-seq data. Once RNA-seq reads have been aligned to the reference genome it is possible to determine the number of aligned reads overlapping each base-pair resulting in the genome base-pair coverage curve as shown in Figure 1. In the example shown in Figure 1, most of the reads overlap known exons from a gene. Those reads can be used to compute a count matrix at the exon or gene feature levels. Some reads span exon-exon junctions (jx) and while most match the annotation, some do not (jx 3 and 4). An exon-exon junction count matrix can be used to identify differentially expressed junctions, which can show which isoforms are differentially expressed given sufficient coverage. For example, junctions 2 and 5 are unique to isoform 2, while junction 6 is unique to isoform 1. The genome base-pair coverage data can be used with derfinder ^8^ to identify expressed regions; some of these could be unannotated exons, which together with the exon-exon junction data could help establish new isoforms.

**Figure 2.**
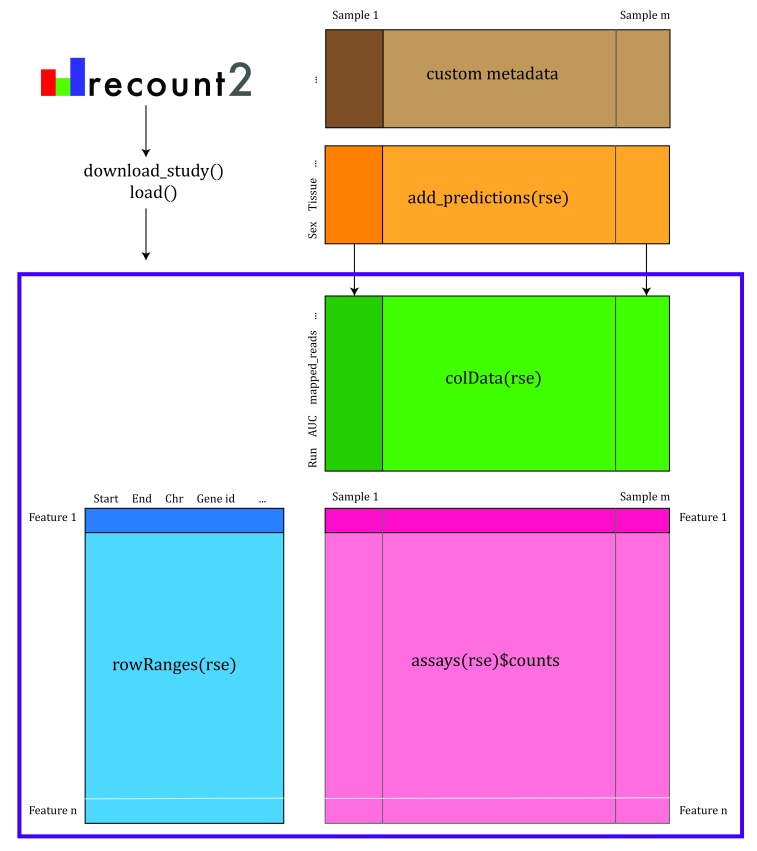
recount2 provides coverage count matrices in RangedSummarizedExperiment (rse) objects. Once the rse object has been downloaded and loaded into R, the feature information is accessed with rowRanges(rse) (blue box), the counts with assays(rse)$counts (pink box) and the sample metadata with colData(rse) (green box). The sample metadata can be expanded using add_predictions(rse) (orange box) or with custom code (brown box) matching by a unique sample identifier such as the SRA Run ID. The rse object is inside the purple box and matching data is highlighted in each box.

recount2 provides gene, exon, and exon-exon junction count matrices both in text format and *RangedSummarizedExperiment* objects (rse) ^9^ as shown in Figure 2. These rse objects provide information about the expression features (for example gene IDs) and the samples. In this workflow we will explain how to add metadata to the rse objects in recount2 in order to ask biological questions. recount2 also provides coverage data in the form of bigWig files. All four features can be accessed with the recount Bioconductor package ^7^. recount also allows sending queries to snaptron ^10^ to search for specific exon-exon junctions.

### Packages used in the workflow

In this workflow we will use several Bioconductor packages. To reproduce the entirety of this workflow, install the packages using the following code after installing R 3.4.x from CRAN in order to use Bioconductor version 3.5 or newer.



## Install packages from Bioconductor
source("https://bioconductor.org/biocLite.R")
biocLite(c("recount","GenomicRanges","limma","edgeR","DESeq2",
	"regionReport","clusterProfiler","org.Hs.eg.db","gplots","derfinder",
	"rtracklayer","GenomicFeatures","bumphunter","derfinderPlot",
	"devtools"))
                    


Once they are installed, load all the packages with the following code.



library("recount")
library("GenomicRanges")
library("limma")
library("edgeR")
library("DESeq2")
library("regionReport")
library("clusterProfiler")
library("org.Hs.eg.db")
library("gplots")
library("derfinder")
library("rtracklayer")
library("GenomicFeatures")
library("bumphunter")
library("derfinderPlot")
library("devtools")



### Coverage counts provided by recount2

**Figure 3.**
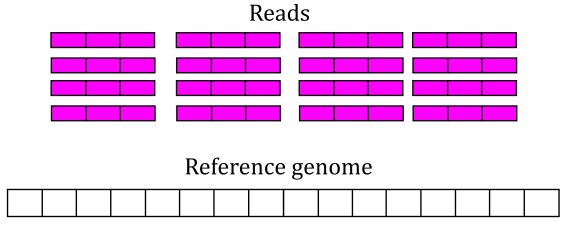
RNA-seq starting data. 16 RNA-seq un-aligned RNA-seq reads 3 base-pairs long are shown (pink boxes) alongside a reference genome that is 16 base-pairs long (white box).

The most accessible features are the gene, exon and exon-exon junction count matrices. This section explains them in greater detail. Figure 3 shows 16 RNA-seq reads, each 3 base-pairs long, and a reference genome.

**Figure 4.**
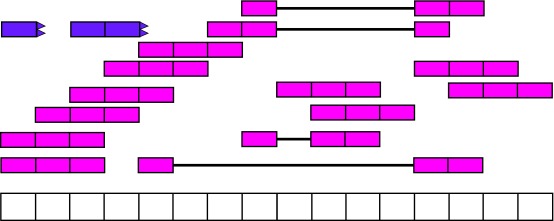
Aligned RNA-seq reads. Spice-aware RNA-seq aligners such as Rail-RNA are able to find the coordinates to which the reads map, even if they span exon-exon junctions (connected boxes). Rail-RNA soft clips some reads (purple boxes with rough edges) such that a portion of these reads align to the reference genome.

Reads in the recount2 resource were aligned with the splice-aware Rail-RNA aligner ^11^. Figure 4 shows the reads aligned to the reference genome. Some of the reads are split as they span an exon-exon junction. Two of the reads were soft clipped meaning that just a portion of the reads aligned (top left in purple).

**Figure 5.**
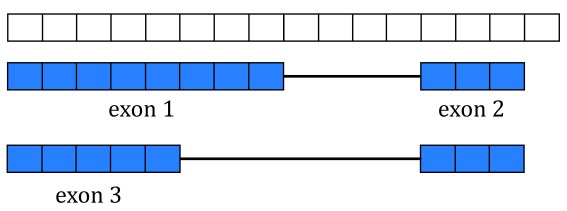
Gene annotation. A single gene with two isoforms composed by three distinct exons (blue boxes) is illustrated. Exons 1 and 3 share the first five base-pairs while exon 2 is common to both isoforms.

In order to compute the gene and exon count matrices we first have to process the annotation, which for recount2 is Gencode v25 (CHR regions) with hg38 coordinates. Although recount can generate count matrices for other annotations using hg38 coordinates. Figure 5 shows two isoforms for a gene composed of 3 different exons.

**Figure 6.**
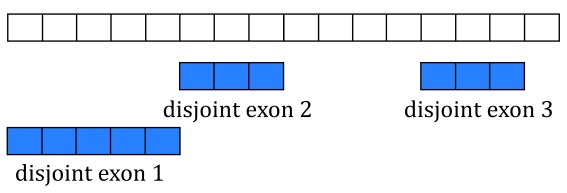
Disjoint exons. Windows of distinct exonic sequence for the example gene. Disjoint exons 1 and 2 form exon 1.



The coverage curve is at base-pair resolution so if we are interested in gene counts we have to be careful not to double count base-pairs 1 through 5 that are shared by exons 1 and 3 ( Figure 5). Using the function disjoin() from GenomicRanges ^12^ we identified the distinct exonic sequences (disjoint exons). The following code defines the exon coordinates that match Figure 5 and the resulting disjoint exons for our example gene. The resulting disjoint exons are shown in Figure 6.

library("GenomicRanges")
exons <-GRanges("seq",IRanges(start = c(1,1,13),end = c(5,8,15)))
exons

## GRanges object with 3 ranges and 0 metadata columns:
##       seqnames    ranges strand
##          <Rle> <IRanges>  <Rle>
##   [1]      seq  [ 1,  5]      *
##   [2]      seq  [ 1,  8]      *
##   [3]      seq  [13, 15]      *
##   -------				
##   seqinfo: 1 sequence from an unspecified genome; no seqlengths

disjoin(exons)

## GRanges object with 3 ranges and 0 metadata  columns:
##       seqnames    ranges strand
##          <Rle> <IRanges>  <Rle>
##   [1]      seq  [ 1,  5]      *
##   [2]      seq  [ 1,  8]      *
##   [3]      seq  [13, 15]      *
##   -------				
##   seqinfo: 1 sequence from an unspecified genome; no seqlengths



**Figure 7.**
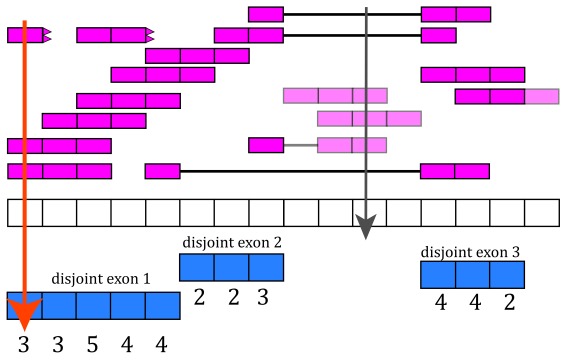
Base-pair coverage counting for exonic base-pairs. At each exonic base-pair we compute the number of reads overlapping that given base-pair. The first base (orange arrow) has 3 reads overlapping that base-pair. Base-pair 11 has a coverage of 3 but does not overlap known exonic sequence, so that information is not used for the gene and exon count matrices (grey arrow). If a read partially overlaps exonic sequence, only the portion that overlaps is used in the computation (see right most read).

Now that we have disjoint exons, we can compute the base-pair coverage for each of them as shown in Figure 7. That is, for each base-pair that corresponds to exonic sequence, we compute the number of reads overlapping that given base-pair. For example, the first base-pair is covered by 3 different reads and it does not matter whether the reads themselves were soft clipped. Not all reads or bases of a read contribute information to this step, as some do not overlap known exonic sequence (light pink in Figure 7).

**Figure 8.**
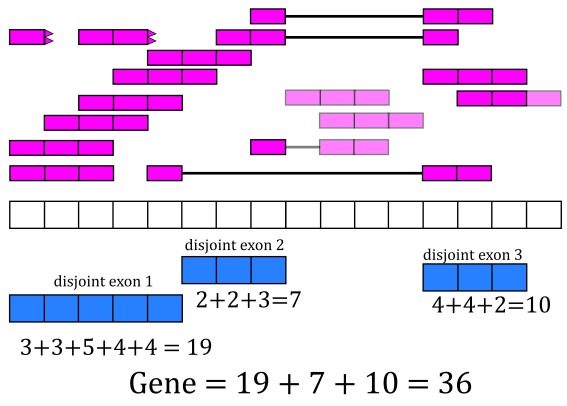
Exon and gene coverage counts. The coverage counts for each disjoint exon are the sum of the base-pair coverage. The gene coverage count is the sum of the disjoint exons coverage counts.

With base-pair coverage for the exonic sequences computed, the coverage count for each distinct exon is simply the sum of the base-pair coverage for each base in a given distinct exon. For example, the coverage count for disjoint exon 2 is 2 + 2 + 3 = 7 as shown in Figure 8. The gene coverage count is then $\sum_{i}^{n}$ coverage *_i_* where *n* is the number of exonic base-pairs for the gene and is equal to the sum of the coverage counts for its disjoint exons as shown in Figure 8.

For the exons, recount2 provides the disjoint exons coverage count matrix. It is possible to reconstruct the exon coverage count matrix by summing the coverage count for the disjoint exons that compose each exon. For example, the coverage count for exon 1 would be the sum of the coverage counts for disjoint exons 1 and 2, that is 19 + 7 = 26. Some methods might assume that double counting of the shared base-pairs was performed while others assume or recommend the opposite.

### Scaling coverage counts

The coverage counts described previously are the ones actually included in the rse objects in recount2 instead of typical read count matrices. This is an important difference to keep in mind as most methods were developed for read count matrices. Part of the sample metadata available from recount2 includes the read length and number of mapped reads. Given a target library size (40 million reads by default), the coverage counts in recount2 can be scaled to read counts for a given library size as shown in Equation (1). Note that the resulting scaled read counts are not necessarily integers so it might be necessary to round them if a differential expression (DE) method assumes integer data.

$$\frac{\sum_{i}^{n}{\text{coverage}}_{i}}{\text{Read Length}}*\frac{\mathrm{target}}{\mathrm{mapped}}=\mathrm{scaled}\;\mathrm{read}\;\mathrm{counts}\;(1)$$

**Figure 9.**
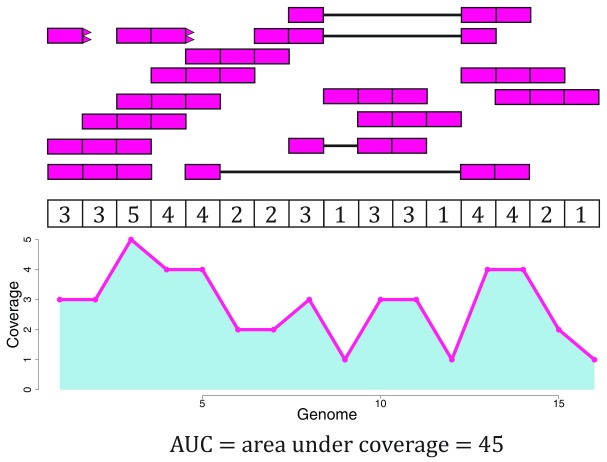
Area under coverage (AUC). The area under coverage is the sum of the base-pair coverage for all positions in the genome regardless of the annotation. It is the area under the base-level coverage curve shown as the light blue area under the pink curve.

From Figure 4 we know that Rail-RNA soft clipped some reads, so a more precise measure than the denominator of Equation (1) is the area under coverage (AUC) which is the sum of the coverage for all base-pairs of the genome, regardless of the annotation as shown in Figure 9. Without soft clipping reads, the AUC would be equal to the number of reads mapped multiplied by the read length. So for our example gene, the scaled counts for a library size of 20 reads would be $\frac{36}{45}*20=16$ and in general calculated with Equation (2). The following code shows how to compute the AUC given a set of aligned reads and reproduce a portion of Figure 9.

$$\frac{\sum_{i}^{n}{\mathrm{coverage}}_{i}}{\mathrm{AUC}}*\mathrm{target}=\mathrm{scaled}\;\mathrm{read}\;\mathrm{counts}\;(2)$$

## Take the example and translate it to R code
library("GenomicRanges")
reads <-GRanges("seq",IRanges(
    start = rep(
	c(1,2,3,4,5,7,8,9,10,13,14),
	c(3,1,2,1,2,1,2,1,2,4,1)
    ), width = rep(
        c(1,3,2,3,1,2,1,3,2,3,2,1,3),
        c(1,4,1,2,1,1,2,2,1,1, 2,1,1)
    )
))
## Get the base-level genome coverage curve
cov <-as.integer(coverage(reads)$seq)

## AUC
sum(cov)
                    




## [1] 45

## Code for reproducing the bottom portion of Figure 8.
pdf("base_pair_coverage.pdf",width =20)
par(mar = c(5,6,4,2)+0.1)
plot(cov,type ="o",col ="violetred1",lwd =10,ylim = c(0,5),
     xlab ="Genome",ylab ="Coverage",cex.axis =2,cex.lab =3,
     bty ="n")
polygon(c(1,seq_len(length(cov)),length(cov)),c(0,cov,0),         
		border =NA,density =-1,col ="light blue")
points(seq_len(length(cov)),cov,col ="violetred1",type ="o",        
        lwd =10)
dev.off()



The recount function scale_counts() computes the scaled read counts for a target library size of 40 million reads and we highly recommend using it before doing other analyses. The following code shows how to use scale_counts() and that the resulting read counts per sample can be lower than the target size (40 million). This happens when not all mapped reads overlap known exonic base-pairs of the genome. In our example, the gene has a scaled count of 16 reads for a library size of 20 reads, meaning that 4 reads did not overlap exonic sequences.



## Check that the number of reads is less than or equal to 40 million 
## after scaling.
library("recount")
rse_scaled <-scale_counts(rse_gene_SRP009615,round =FALSE)
summary(colSums(assays(rse_scaled)$counts))/1e6

##    Min. 1st Qu.  Median    Mean 3rd Qu.    Max.
##   22.62   29.97   34.00   31.96   34.86   36.78



### Enriching the annotation

**Figure 10.**
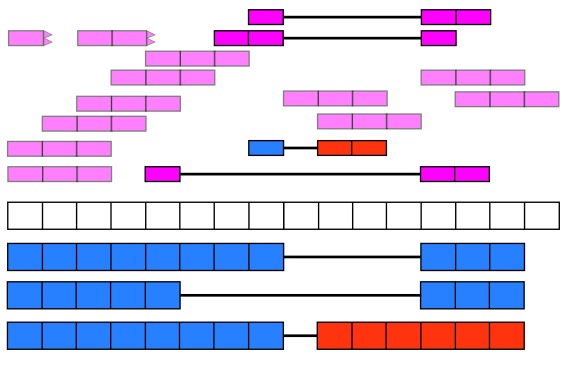
Exon-exon junctions go beyond the annotation. Reads spanning exon-exon junctions are highlighted and compared against the annotation. Three of them match the annotated junctions, but one (blue and orange read) spans an unannotated exon-exon junction with the left end matching the annotation and the right end hinting at a possible new isoform for this gene (blue and orange isoform).

Data in recount2 can be used for annotation-agnostic analyses and enriching the known annotation. Just like exon and gene coverage count matrices, recount2 provides exon-exon junction count matrices. These matrices can be used to identify new isoforms ( Figure 10) or identify differentially expressed isoforms. For example, exon-exon junctions 2, 5 and 6 in Figure 1 are only present in one annotated isoform. Snaptron ^10^ allows programatic and high-level queries of the exon-exon junction information and its graphical user interface is specially useful for visualizing this data. Inside R, the recount function snaptron_query() can be used for searching specific exon-exon junctions in recount2.

**Figure 11.**
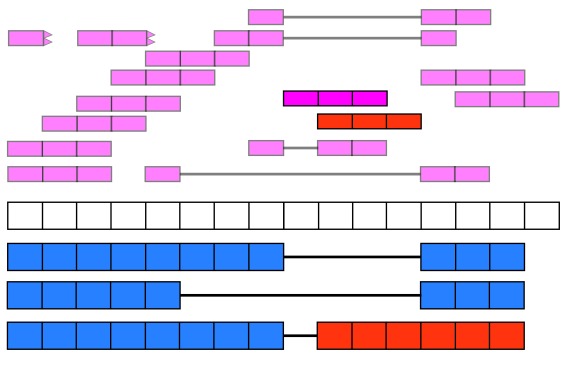
Intron retention events. Some reads might align with known intronic segments of the genome and provide information for exploring intron retention events (pink read). Some might support an intron retention event or a new isoform when coupled with exon-exon junction data (orange read).

**Figure 12.**
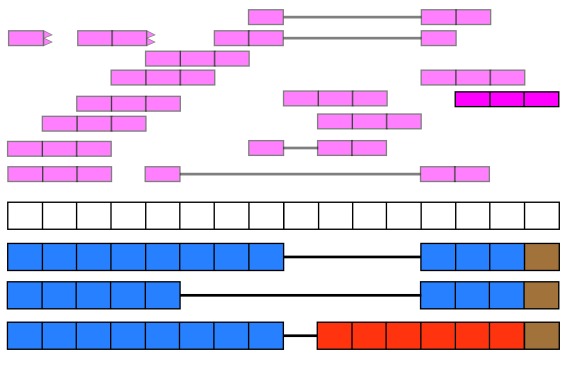
Exon boundaries. Reads that go beyond the known exon boundaries can inform us of whether the annotated boundaries are correct or if there was a run-off transcription event.

The base-pair coverage data from recount2 can be used together with derfinder ^8^ to identify expressed regions of the genome, providing another annotation-agnostic analysis of the expression data. Using the function expressed_regions() we can identify regions of expression based on a given data set in recount2. These regions might overlap known exons but can also provide information about intron retention events ( Figure 11), improve detection of exon boundaries ( Figure 12), and help identify new exons ( Figure 1) or expressed sequences in intergenic regions. Using coverage_matrix() we can compute a coverage matrix based on the expressed regions or another set of genomic intervals. The resulting matrix can then be used for a DE analysis, just like the exon, gene and exon-exon junction matrices.

### Gene level analysis

Having reviewed how the coverage counts in recount2 were produced, we can now do a DE analysis. We will use data from 72 individuals spanning the human lifespan, split into 6 age groups with SRA accession SRP045638 ^13^. The function download_study() requires a SRA accession which can be found using abstract_search(). download_study() can then be used to download the gene coverage count data as well as other expression features. The files are saved in a directory named after the SRA accession, in this case SRP045638.



library("recount")

## Find the project ID by searching abstracts of studies
abstract_search("human brain development by age")






                        
## number_samples species
## 1296             72	 human
##                                                                             abstract
## 1296 RNAseq data of 36 samples across human brain development by age group from LIBD
##	  project
## 1296 SRP045638

## Download the data if it is not there
if(!file.exists(file.path("SRP045638","rse_gene.Rdata"))) {
    download_study("SRP045638", type ="rse-gene")
}

## 2017-07-30 10:11:16 downloading file rse_gene.Rdata to SRP045638

## Check that the file was downloaded
file.exists(file.path("SRP045638","rse_gene.Rdata"))

## [1] TRUE

## Load the data
load(file.path("SRP045638","rse_gene.Rdata"))



The coverage count matrices are provided as *RangedSummarizedExperiment* objects (rse) ^9^. These objects store information at the feature level, the samples and the actual count matrix as shown in Figure 1 of Love *et al.*, 2016 ^3^. Figure 2 shows the actual rse objects provided by recount2 and how to access the different portions of the data. Using a unique sample ID such as the SRA Run ID it is possible to expand the sample metadata. This can be done using the predicted phenotype provided by add_predictions() ^14^, pulling information from GEO via find_geo() and geo_characteristics(), or with custom code.

### Metadata

Using the colData() function we can access sample metadata. More information on these metadata is provided in the Supplementary material of the recount2 paper ^7^, and we provide a brief review here. The rse objects for SRA data sets include 21 columns with mostly technical information. The GTEx and TCGA rse objects include additional metadata as available from the raw sources. In particular, we compiled metadata for GTEx using the v6 phenotype information available at gtexportal.org, and we put together a large table of TCGA case and sample information by combining information accumulated across Seven Bridges’ Cancer Genomics Cloud and TCGAbiolinks ^15^.



## One row per sample, one column per phenotype variable
dim(colData(rse_gene))

## [1] 72 21

## Mostly technical variables are included
colnames(colData(rse_gene))

##  [1] "project"
##  [2] "sample"
##  [3] "experiment"
##  [4] "run"
##  [5] "read_count_as_reported_by_sra"
##  [6] "reads_downloaded"
##  [7] "proportion_of_reads_reported_by_sra_downloaded"
##  [8] "paired_end"
##  [9] "sra_misreported_paired_end"
## [10] "mapped_read_count"
## [11] "auc"
## [12] "sharq_beta_tissue"
## [13] "sharq_beta_cell_type"
## [14] "biosample_submission_date"
## [15] "biosample_publication_date"
## [16] "biosample_update_date"
## [17] "avg_read_length"
## [18] "geo_accession"
## [19] "bigwig_file"
## [20] "title"
## [21] "characteristics"




**Technical variables** Several of these technical variables include the number of reads as reported by SRA, the actual number of reads Rail-RNA was able to download (which might be lower in some cases), the number of reads mapped by Rail-RNA, whether the sample is paired-end or not, the coverage AUC and the average read length (times 2 for paired-end samples). Note that the sample with SRA Run ID SRR2071341 has about 240.8 million reads as reported by SRA, while it has 120.4 million spots reported in https://trace.ncbi.nlm.nih.gov/Traces/sra/?run=SRR2071341; that is because it is a paired-end sample (2 reads per spot). These details are important for those interested in writing alternative scaling functions to scale_counts().



## Input reads: number reported by SRA might be larger than number
## of reads Rail-RNA downloaded
colData(rse_gene)[,c("read_count_as_reported_by_sra","reads_downloaded")]

## DataFrame with 72 rows and 2 columns
##	      read_count_as_reported_by_sra reads_downloaded
##	                          <integer>	   <integer>
## SRR2071341                     240797206        240797206
## SRR2071345                      82266652         82266652
## SRR2071346                     132911310        132911310
## SRR2071347                      74051302         74051302
## SRR2071348                     250259914        250259914
## ...                                  ...              ...
## SRR1554541                     186250218        162403466
## SRR1554554                     140038024        121793680
## SRR1554535                     106244496         91185969
## SRR1554558                     200687480        170754145
## SRR1554553                      90579486         51803404

summary(colData(rse_gene)$proportion_of_reads_reported_by_sra_downloaded)

##   Min. 1st Qu. Median   Mean 3rd Qu.   Max.
## 0.5719  0.9165 0.9788 0.9532	 1.0000	1.0000

## AUC information used by scale_counts() by default
head(colData(rse_gene)$auc)

## [1] 22950214241  7553726235 12018044330  7041243857 24062460144 45169026301

## Alternatively, scale_scounts() can use the number of mapped reads
## and other information
colData(rse_gene)[,c("mapped_read_count","paired_end","avg_read_length")]

## DataFrame with 72 rows and 3 columns
##	      mapped_read_count paired_end avg_read_length
##                    <integer>	 <logical>	 <integer>
## SRR2071341	      232970536       TRUE	       200
## SRR2071345	       78431778       TRUE	       200
## SRR2071346	      124493632       TRUE	       200
## SRR2071347	       71742875       TRUE	       200
## SRR2071348	      242992735       TRUE	       200
## ...	                    ...	       ...	       ...
## SRR1554541	      162329325       TRUE	       174
## SRR1554554	      121738246       TRUE	       173
## SRR1554535	       91120421       TRUE	       171
## SRR1554558	      170648458       TRUE	       170
## SRR1554553	       51684462       TRUE	       114



**Biological information** Other metadata variables included provide more biological information, such as the SHARQ beta tissue and cell type predictions, which are based on processing the abstract of papers. This information is available for some of the SRA projects.




## SHARQ tissue predictions: not present for all studies
head(colData(rse_gene)$sharq_beta_tissue)

## [1] NA NA NA NA NA NA

head(colData(rse_gene_SRP009615)$sharq_beta_tissue)

## [1] "blood" "blood" "blood" "blood" "blood" "blood"



For some data sets we were able to find the GEO accession IDs, which we then used to create the title and characteristics variables. If present, the characteristics information can be used to create additional metadata variables by parsing the CharacterList in which it is stored. Since the input is free text, sometimes more than one type of wording is used to describe the same information, meaning that we might have to process that information in order to build a more convenient variable, such as a factor vector.



## GEO information was absent for the SRP045638 data set
colData(rse_gene)[,c("geo_accession","title","characteristics")]

## DataFrame with 72 rows and 3 columns
##	      geo_accession	  title  characteristics
##	        <character> <character>  <CharacterList>
## SRR2071341	         NA	     NA	              NA
## SRR2071345	         NA	     NA	              NA
## SRR2071346	         NA	     NA	              NA
## SRR2071347	         NA	     NA	              NA
## SRR2071348	         NA	     NA	              NA
## ...	                ...	    ...	             ...
## SRR1554541	         NA	     NA	              NA
## SRR1554554	         NA	     NA	              NA
## SRR1554535	         NA	     NA	              NA
## SRR1554558	         NA	     NA	              NA
## SRR1554553	         NA	     NA	              NA

## GEO information for the SRP009615 data set
head(colData(rse_gene_SRP009615)$geo_accession)

## [1] "GSM836270" "GSM836271" "GSM836272" "GSM836273" "GSM847561" "GSM847562"

head(colData(rse_gene_SRP009615)$title,2)

## [1] "K562 cells with shRNA targeting SRF gene cultured with no
## doxycycline (uninduced - UI), rep1."
## [2] "K562 cells with shRNA targeting SRF gene cultured with
## doxycycline for 48 hours (48 hr), rep1."

head(colData(rse_gene_SRP009615)$characteristics,2)

## CharacterList of length 2
## [[1]] cells: K562 shRNA expression: no treatment: Puromycin
## [[2]] cells: K562 shRNA expression: yes, targeting SRF treatment: Puromycin, doxycycline

## Similar but not exactly the same wording used for two different samples
colData(rse_gene_SRP009615)$characteristics[[1]]

## [1] "cells: K562"          "shRNA expression: no" "treatment: Puromycin"





colData(rse_gene_SRP009615)$characteristics[[11]]
                    




## [1] "cell line: K562"
## [2] "shRNA expression: no shRNA expression"
## [3] "treatment: Puromycin"
                    




## Extract the target information
target <- sapply(colData(rse_gene_SRP009615)$characteristics,"[",2)
target
                    




##  [1] "shRNA expression: no"
##  [2] "shRNA expression: yes, targeting SRF"
##  [3] "shRNA expression: no"
##  [4] "shRNA expression: yes targeting SRF"
##  [5] "shRNA expression: no shRNA expression"
##  [6] "shRNA expression: expressing shRNA targeting EGR1"
##  [7] "shRNA expression: no shRNA expression"
##  [8] "shRNA expression: expressing shRNA targeting EGR1"
##  [9] "shRNA expression: no shRNA expression"
## [10] "shRNA expression: expressing shRNA targeting ATF3"
## [11] "shRNA expression: no shRNA expression"
## [12] "shRNA expression: expressing shRNA targeting ATF3"
                    




## Build a useful factor vector, set the reference level and append the result
## to the colData() slot
target_factor <-sapply(strsplit(target,"targeting "),"[",2) target_factor[is.na(target_factor)] <-"none"
target_factor <-factor(target_factor)
target_factor <-relevel(target_factor,"none")
target_factor
                    




##  [1] none SRF  none SRF none EGR1 none EGR1 none ATF3 none ATF3
## Levels: none ATF3 EGR1 SRF
                    




colData(rse_gene_SRP009615)$target_factor <- target_factor
                    


As shown in Figure 2, we can expand the biological metadata information by adding predictions based on RNA-seq data ^14^. The predictions include information about sex, sample source (cell line vs tissue), tissue and the sequencing strategy used. To add the predictions, simply use the function add_predictions() to expand the colData() slot.



## Before adding predictions
dim(colData(rse_gene))
                    




## [1] 72 21
                    




## Add the predictions
rse_gene <-add_predictions(rse_gene)
                    




## 2017-07-30 10:11:20 downloading the predictions to
## /var/folders/cx/n9s558kx6fb7jf5z_pgszgb80000gn/T//RtmpLufhkr/PredictedPhenotypes_v0.0.03.rda
                    




## After adding the predictions
dim(colData(rse_gene))
                    




## [1] 72 33
                    




## Explore the variables
colData(rse_gene)[,22:ncol(colData(rse_gene))]
                    




## DataFrame with 72 rows and 12 columns
##            reported_sex predicted_sex accuracy_sex reported_samplesource
##                <factor>      <factor>    <numeric>              <factor>
## SRR2071341       female        female    0.8428571                    NA
## SRR2071345         male          male    0.8428571                    NA
## SRR2071346         male          male    0.8428571                    NA
## SRR2071347       female        female    0.8428571                    NA
## SRR2071348       female        female    0.8428571                    NA
## ...                 ...           ...          ...                   ...
## SRR1554541         male        female    0.8428571                    NA
## SRR1554554       female        female    0.8428571                    NA
## SRR1554535         male          male    0.8428571                    NA
## SRR1554558       female        female    0.8428571                    NA
## SRR1554553         male          male    0.8428571                    NA
##            predicted_samplesource accuracy_samplesource reported_tissue
##                          <factor>             <numeric>        <factor>
## SRR2071341                 tissue                    NA              NA
## SRR2071345                 tissue             0.8923497              NA
## SRR2071346                 tissue                    NA              NA
## SRR2071347                 tissue                    NA              NA
## SRR2071348                 tissue                    NA              NA
## ...                           ...                   ...             ...
## SRR1554541                 tissue                    NA              NA
## SRR1554554                 tissue                    NA              NA
## SRR1554535                 tissue                    NA              NA
## SRR1554558                 tissue                    NA              NA
## SRR1554553                 tissue             0.8923497              NA
##            predicted_tissue accuracy_tissue reported_sequencingstrategy
##                    <factor>       <numeric>                    <factor>
## SRR2071341            Brain       0.4707854                      PAIRED
## SRR2071345            Brain       0.4707854                      PAIRED
## SRR2071346            Brain       0.4707854                      PAIRED
## SRR2071347            Brain       0.4707854                      PAIRED
## SRR2071348            Brain       0.4707854                      PAIRED
## ...                     ...             ...                         ...
## SRR1554541            Brain       0.4707854                      PAIRED
## SRR1554554            Brain       0.4707854                      PAIRED
## SRR1554535            Brain       0.4707854                      PAIRED
## SRR1554558            Brain       0.4707854                      PAIRED
## SRR1554553            Brain       0.4707854                      PAIRED
##            predicted_sequencingstrategy accuracy_sequencingstrategy
##                                <factor>                   <numeric>
## SRR2071341                       PAIRED                   0.8915381
## SRR2071345                       PAIRED                   0.8915381
## SRR2071346                       PAIRED                   0.8915381
## SRR2071347                       PAIRED                   0.8915381
## SRR2071348                       PAIRED                   0.8915381
## ...                                 ...                         ...
## SRR1554541                       PAIRED                   0.8915381
## SRR1554554                       PAIRED                   0.8915381
## SRR1554535                       PAIRED                   0.8915381
## SRR1554558                       PAIRED                   0.8915381
## SRR1554553                       PAIRED                   0.8915381
                    


**Adding more information** Ultimately, more sample metadata information could be available elsewhere, which can be useful for analyses. This information might be provided in the paper describing the data, the SRA Run Selector or other sources. As shown in Figure 2, it is possible to append information to the colData() slot as long as there is a unique sample identifier such as the SRA Run ID.

For our example use case, project SRP045638 has a few extra biologically relevant variables via the SRA Run selector https://trace.ncbi.nlm.nih.gov/Traces/study/?acc=SRP045638. We can download that information into text file named SraRunTable.txt by default, then load it into R, sort it appropriately and then append it to the colData() slot. Below we do so for the SRP045638 project.



## Save the information from
## https://trace.ncbi.nlm.nih.gov/Traces/study/?acc=SRP045638
## to a table. We saved the file as SRP045638/SraRunTable.txt.
file.exists(file.path("SRP045638","SraRunTable.txt"))
                    




## [1] TRUE
                    




## Read the table
sra <-read.table(file.path("SRP045638","SraRunTable.txt"),
    header=TRUE,sep="\t")
     
## Explore it
head(sra)
                    




                        ##   AssemblyName_s AvgSpotLen_l  BioSample_s Experiment_s
## 1         GRCh37          179 SAMN02731372    SRX683791
## 2         GRCh37          179 SAMN02731373    SRX683792
## 3         GRCh37          171 SAMN02999518    SRX683793
## 4         GRCh37          184 SAMN02999519    SRX683794
## 5         GRCh37          182 SAMN02999520    SRX683795
## 6         GRCh37          185 SAMN02999521    SRX683796
##                   Library_Name_s LoadDate_s MBases_l MBytes_l RIN_s
## 1 R2835_DLPFC_polyA_RNAseq_total 2014-08-21     6452     3571   8.3
## 2 R2857_DLPFC_polyA_RNAseq_total 2014-08-21     6062     2879   8.4
## 3 R2869_DLPFC_polyA_RNAseq_total 2014-08-21     8696     4963   8.7
## 4 R3098_DLPFC_polyA_RNAseq_total 2014-08-21     4479     2643   5.3
## 5 R3452_DLPFC_polyA_RNAseq_total 2014-08-21    11634     6185   9.6
## 6 R3462_DLPFC_polyA_RNAseq_total 2014-08-21    14050     7157   6.4
##   ReleaseDate_s      Run_s SRA_Sample_s Sample_Name_s   age_s disease_s
## 1    2014-11-13 SRR1554533    SRS686961   R2835_DLPFC 67.7800   Control
## 2    2014-11-13 SRR1554534    SRS686962   R2857_DLPFC 40.4200   Control
## 3    2014-11-13 SRR1554535    SRS686963   R2869_DLPFC 41.5800   control
## 4    2014-11-13 SRR1554536    SRS686964   R3098_DLPFC 44.1700   control
## 5    2014-11-13 SRR1554537    SRS686965   R3452_DLPFC -0.3836   control
## 6    2014-11-13 SRR1554538    SRS686966   R3462_DLPFC -0.4027   control
##   isolate_s race_s  sex_s Assay_Type_s BioProject_s BioSampleModel_s
## 1     DLPFC     AA female      RNA-Seq  PRJNA245228            Human
## 2     DLPFC     AA   male      RNA-Seq  PRJNA245228            Human
## 3     R2869     AA   male      RNA-Seq  PRJNA245228            Human
## 4     R3098     AA female      RNA-Seq  PRJNA245228            Human
## 5     R3452     AA female      RNA-Seq  PRJNA245228            Human
## 6     R3462     AA female      RNA-Seq  PRJNA245228            Human
##   Consent_s Fraction_s InsertSize_l        Instrument_s LibraryLayout_s
## 1    public      total            0 Illumina HiSeq 2000          PAIRED
## 2    public      total            0 Illumina HiSeq 2000          PAIRED
## 3    public      total            0 Illumina HiSeq 2000          PAIRED
## 4    public      total            0 Illumina HiSeq 2000          PAIRED
## 5    public      total            0 Illumina HiSeq 2000          PAIRED
## 6    public      total            0 Illumina HiSeq 2000          PAIRED
##   LibrarySelection_s LibrarySource_s   Organism_s Platform_s SRA_Study_s
## 1               cDNA  TRANSCRIPTOMIC Homo sapiens   ILLUMINA   SRP045638
## 2               cDNA  TRANSCRIPTOMIC Homo sapiens   ILLUMINA   SRP045638
## 3               cDNA  TRANSCRIPTOMIC Homo sapiens   ILLUMINA   SRP045638
## 4               cDNA  TRANSCRIPTOMIC Homo sapiens   ILLUMINA   SRP045638
## 5               cDNA  TRANSCRIPTOMIC Homo sapiens   ILLUMINA   SRP045638
## 6               cDNA  TRANSCRIPTOMIC Homo sapiens   ILLUMINA   SRP045638
##   biomaterial_provider_s tissue_s
## 1                   LIBD    DLPFC
## 2                   LIBD    DLPFC
## 3                   LIBD    DLPFC
## 4                   LIBD    DLPFC
## 5                   LIBD    DLPFC
## 6                   LIBD    DLPFC
                    




## We will remove some trailing ’_s’ from the variable names
colnames(sra) <-gsub("_s$","",colnames(sra))


## Choose some variables we want to add
sra_vars <-c("sex","race","RIN","age","isolate","disease","tissue")

## Re-organize the SRA table based on the SRA Run IDs we have
sra <- sra[match(colData(rse_gene)$run, sra$Run), ]

## Double check the order
identical(colData(rse_gene)$run,as.character(sra$Run))
                    




## [1] TRUE
                    




## Append the variables of interest
colData(rse_gene) <-cbind(colData(rse_gene), sra[, sra_vars])

## Final dimensions
dim(colData(rse_gene))
                    




## [1] 72 40
                    




## Explore result
colData(rse_gene)[,34:ncol(colData(rse_gene))]
                    




## DataFrame with 72 rows and 7 columns
## 		   sex 	   race       RIN 	age  isolate  disease
## 	      <factor> <factor> <numeric> <numeric> <factor> <factor>
## SRR2071341   female 	     AA       8.3   67.7800    DLPFC  Control
## SRR2071345     male 	     AA       8.4   40.4200    DLPFC  Control
## SRR2071346     male 	     AA       8.7   41.5800    R2869  control
## SRR2071347   female 	     AA       5.3   44.1700    R3098  control
## SRR2071348   female 	     AA       9.6   -0.3836    R3452  control
## ... 		   ... 	    ...       ...       ...      ...      ...
## SRR1554541     male 	     AA       5.7   -0.3836    R3485  control
## SRR1554554   female 	     AA       8.1    0.3041    R3669  control
## SRR1554535     male 	     AA       8.7   41.5800    R2869  control
## SRR1554558   female     CAUC       9.1   16.7000    R4028  control
## SRR1554553     male     CAUC       8.4    0.3918    R3652  control
## 		tissue
## 	      <factor>
## SRR2071341    DLPFC
## SRR2071345    DLPFC
## SRR2071346    DLPFC
## SRR2071347    DLPFC
## SRR2071348    DLPFC
## ... 		   ...
## SRR1554541    DLPFC
## SRR1554554    DLPFC
## SRR1554535    DLPFC
## SRR1554558    DLPFC
## SRR1554553    DLPFC
                    


Since we have the predicted sex as well as the reported sex via the SRA Run Selector, we can check whether they match.



table("Predicted"=colData(rse_gene)$predicted_sex,
    "Observed"=colData(rse_gene)$sex)
                    




## 	       Observed
## Predicted    female male
##   female 	    24    8
##   male 	     0   40
##   Unassigned      0    0
                    


### DE setup

Now that we have all the metadata available we can perform a DE analysis. The original study for project SRP045638 ^13^ looked at differences between 6 age groups: prenatal, infant, child, teen, adult and late life. The following code creates these six age groups.



## Create the original 6 age groups
colData(rse_gene)$age_group <-factor(
    ifelse(colData(rse_gene)$age<0,"prenatal",
    ifelse(colData(rse_gene)$age>=0&colData(rse_gene)$age<1"infant",
    ifelse(colData(rse_gene)$age>=1&colData(rse_gene)$age<10,"child",
    ifelse(colData(rse_gene)$age>=10&colData(rse_gene)$age<20,"teen",
    ifelse(colData(rse_gene)$age>=20&colData(rse_gene)$age<50,"adult",
	"late life"))))),
    levels = c("prenatal","infant","child","teen","adult","late life")
)
                    


Most of the DE signal from the original study was between the prenatal and postnatal samples. To simplify the analysis, we will focus on this comparison.



## Create prenatal factor
colData(rse_gene)$prenatal <-factor(
    ifelse(colData(rse_gene)$age_group=="prenatal","prenatal","postnatal"),
    levels = c("prenatal","postnatal"))
                    


As we saw earlier in Figure 9, it is important to scale the coverage counts to read counts. To highlight the fact that we scaled the counts, we will use a new object name and delete the previous one. However, in practice we would simply overwrite the rse object with the output of scale_counts(rse).



## Scale counts
rse_gene_scaled <-scale_counts(rse_gene)

## To highlight that we scaled the counts
rm(rse_gene)
                    


Having scaled the counts, we then filter out genes that are lowly expressed and extract the count matrix.



## Extract counts and filter out lowly expressed geens
counts <-assays(rse_gene_scaled)$counts
filter <-rowMeans(counts)>0.5
                    


### DE analysis

**Figure 13.**
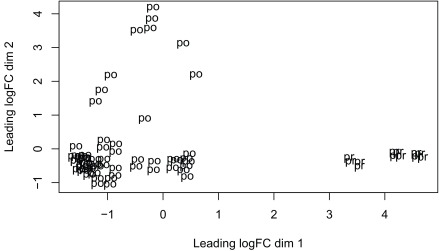
Multi-dimensional scaling plot of the gene level data by age group.

**Figure 14.**
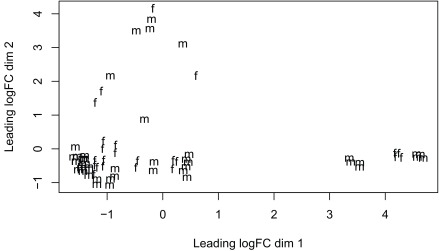
Multi-dimensional scaling plot of the gene level data by sex.

**Figure 15.**
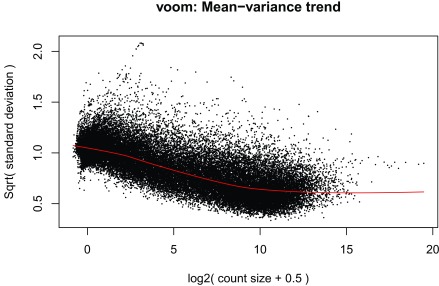
voom mean-variance plot of the gene level data.



Now that we have scaled the counts, there are multiple DE packages we could use, as described elsewhere ^2, 3^. Since we have 12 samples per group, which is a moderate number, we will use limma-voom ^16^ due to its speed. The model we use tests for DE between prenatal and postnatal samples adjusting for sex and RIN, which is a measure of quality of the input sample. We check the data with multi-dimensional scaling plots ( Figure 13 and Figure 14) as well as the mean-variance plot ( Figure 15). In a real use case we might have to explore the results with different models and perform sensitivity analyses.

library("limma")
library("edgeR")

## Build DGEList object
dge <-DGEList(counts =counts[filter, ])

## Calculate normalization factors
dge <-calcNormFactors(dge)

## Explore the data
plotMDS(dge,labels=substr(colData(rse_gene_scaled)$prenatal,1,2) )
                    




plotMDS(dge,labels = substr(colData(rse_gene_scaled)$sex,1,1) )
                    




tapply(colData(rse_gene_scaled)$RIN,colData(rse_gene_scaled)$prenatal, summary)
                    




## $prenatal
##    Min. 1st Qu. Median  Mean 3rd Qu.  Max.
##   5.700   6.400  8.150 7.767   8.600 9.600
##
## $postnatal
##    Min. 1st Qu. Median  Mean 3rd Qu.  Max.
##   5.300   8.100  8.300 8.197   8.700 9.100
                    




## Specify our design matrix
design <-with(colData(rse_gene_scaled),model.matrix(~sex+RIN+prenatal))
                    




## Run voom
v <-voom(dge, design,plot =TRUE)
                    




## Run remaining parts of the DE analysis
fit <-lmFit(v, design)
fit <-eBayes(fit)
                    


**Figure 16.**
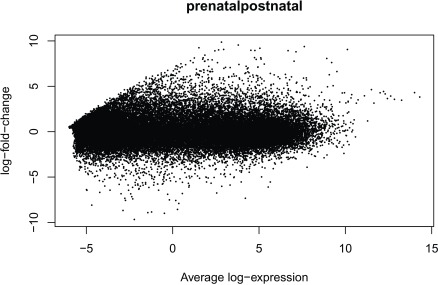
MA plot of the gene level data. Testing for prenatal and postnatal DE adjusting for sex and RIN.

**Figure 17.**
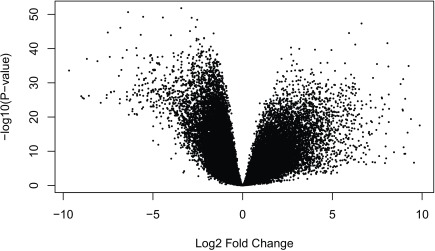
Volcano plot of the gene level data. Testing for prenatal and postnatal DE adjusting for sex and RIN.

Having run the DE analysis, we can explore some of the top results either with an MA plot ( Figure 16) and a volcano plot Figure (17). Both reveal very strong and widespread DE signal.



## Visually explore DE results
limma::plotMA(fit,coef =4)

limma::volcanoplot(fit,coef =4)



### DE report

Now that we have the DE results, we can use some of the tools with the biocView ReportWriting to create a report. One of them is regionReport ^17^, which can create reports from DESeq2 ^18^ and edgeR ^19^ results. It can also handle limma-voom ^16^ results by making them look like DESeq2 results. To do so, we need to extract the relevant information from the limma-voom objects using topTable() and build DESeqDataSet and DESeqResults objects as shown below. A similar conversion is needed to use ideal ^20^, which is another package in the *ReportWriting* biocView category.




## Extract data from limma-voom results
top <-topTable(fit,number =Inf,sort.by ="none",coef ="prenatalpostnatal")

## Build a DESeqDataSet with the count data and model we used
library("DESeq2")
dds <-DESeqDataSet(rse_gene_scaled[filter, ],~sex+RIN+prenatal)

## converting counts to integer mode

## Add gene names keeping only the Ensembl part of the Gencode IDs
rownames(dds) <-gsub("\\..∗","",rownames(dds))

## Build a DESeqResults object with the relevant information
## Note that we are transforming the baseMean so it will look ok
## with DESeq2's plotting functions.
limma_res <-DESeqResults(DataFrame(pvalue =top[,"P.Value"],
	log2FoldChange =top[,"logFC"],
	baseMean = exp(top[,"AveExpr"]),
	padj =top[,"adj.P.Val"]))
rownames(limma_res) <-rownames(dds)

## Specify FDR cutoff to use
metadata(limma_res)[["alpha"]] <-0.001

## Add gene symbols so they will be displayed in the report
limma_res$symbol <-rowRanges(rse_gene_scaled)$symbol[filter]

## Some extra information used by the report function
mcols(dds) <- limma_res
mcols(mcols(dds)) <-DataFrame(type ="results",
    description ="manual incomplete conversion from limma-voom to DESeq2")



Having converted our limma-voom results to DESeq2 results, we can now create the report, which should open automatically in a browser.




library("regionReport")
## This takes about 20 minutes to run
report <-DESeq2Report(dds,project ="SRP045638 gene results with limma-voom",
        output ="gene_report",outdir ="SRP045638",
        intgroup = c("prenatal","sex"),res =limma_res,software ="limma")



If the report doesn’t open automatically, we can open it with browseURL(). A pre-computed version is available as Supplementary File 1.



browseURL(file.path("SRP045638","gene_report.html"))
                    


### GO enrichment

**Figure 18.**
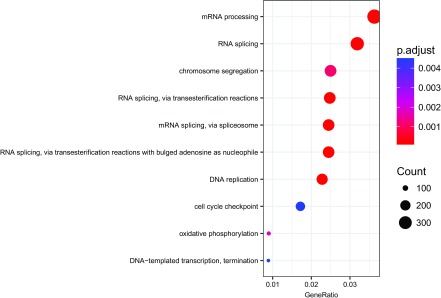
Biological processes enriched in the DE genes.




Using clusterProfiler ^21^ we can then perform several enrichment analyses using the Ensembl gene IDs. Here we show how to perform an enrichment analysis using the biological process ontology ( Figure 18).

library("clusterProfiler")
library("org.Hs.eg.db")


## Remember that limma_res had ENSEMBL IDs for the genes
head(rownames(limma_res))

## [1] "ENSG00000000003" "ENSG00000000005" "ENSG00000000419" "ENSG00000000457"
## [5] "ENSG00000000460" "ENSG00000000938"

## Perform enrichment analysis for Biological Process (BP)
## Note that the argument is keytype instead of keyType in Bioconductor 3.5
enrich_go <-enrichGO(gene = rownames(limma_res)[limma_res$padj<0.001],
    OrgDb =org.Hs.eg.db,keyType ="ENSEMBL",ont ="BP",
    pAdjustMethod ="BH",pvalueCutoff =0.01,qvalueCutoff =0.05,
    universe = rownames(limma_res))

## Visualize enrichment results
dotplot(enrich_go,font.size =7)



Several other analyses can be performed with the resulting list of differentially expressed genes as described previously ^2, 3^, although that is beyond the scope of this workflow.

### Other features

As described in Figure 1, recount2 provides data for expression features beyond genes. In this section we perform a DE analysis using exon data as well as the base-pair resolution information.

### Exon and exon-exon junctions

The exon and exon-exon junction coverage count matrices are similar to the gene level one and can also be downloaded with download_study(). However, these coverage count matrices are much larger than the gene one. Aggressive filtering of lowly expressed exons or exon-exon junctions can reduce the matrix dimensions if this impacts the performance of the DE software used.

**Figure 19.**
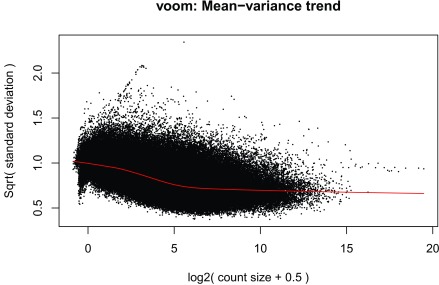
voom mean-variance plot of the exon level data.



Below we repeat the gene level analysis for the disjoint exon data. We first download the exon data, add the expanded metadata we constructed for the gene analysis, explore the data ( Figure 19), and then perform the DE analysis using limma-voom.

## Download the data if it is not there
if(!file.exists(file.path("SRP045638","rse_exon.Rdata"))) {
    download_study("SRP045638",type ="rse-exon")
}
                    




## 2017-07-30 10:37:11 downloading file rse_exon.Rdata to SRP045638
                    




## Load the data
load(file.path("SRP045638","rse_exon.Rdata"))

## Scale and add the metadata (it is in the same order)
identical(colData(rse_exon)$run,colData(rse_gene_scaled)$run)
                    




## [1] TRUE
                    




colData(rse_exon) <-colData(rse_gene_scaled)
rse_exon_scaled <-scale_counts(rse_exon)
## To highlight that we scaled the counts
rm(rse_exon)

## Filter lowly expressed exons
filter_exon <-rowMeans(assays(rse_exon_scaled)$counts)>0.5
round(table(filter_exon)/length(filter_exon)∗100,2)
                    




## filter_exon
## FALSE  TRUE
## 29.08 70.92
                    




## Build DGEList object
dge_exon <-DGEList(counts = assays(rse_exon_scaled)$counts[filter_exon, ])

## Calculate normalization factors
dge_exon <-calcNormFactors(dge_exon)

## Run voom
v_exon <-voom(dge_exon, design,plot =TRUE)
                    




## Run remaining parts of the DE analysis
fit_exon <-lmFit(v_exon, design)
fit_exon <-eBayes(fit_exon)

## Visualize inspect results
limma::volcanoplot(fit_exon,coef =4)
                    




## Get p-values and other statistics
top_exon <-topTable(fit_exon,number =Inf,sort.by ="none",
    coef ="prenatalpostnatal")
table(top_exon$adj.P.Val<0.001)
                    




##
## FALSE  TRUE
## 107303 126075
                    


**Figure 20.**
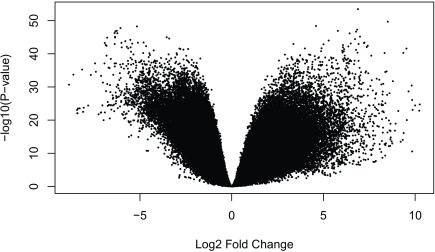
Volcano plot of the exon level data. Testing for prenatal and postnatal DE adjusting for sex and RIN.



Just like at the gene level, we see many exons differentially expressed between prenatal and postnatal samples ( Figure 20). As a first step to integrate the results from the two features, we can compare the list of genes that are differentially expressed versus the genes that have at least one exon differentially expressed.

## Get the gene IDs for genes that are DE at the gene level or that have at
## least one exon with DE signal.
genes_w_de_exon <-unique(rownames(rse_exon_scaled)[top_exon$adj.P.Val<0.001])
genes_de <-rownames(rse_gene_scaled)[which(filter)[top$adj.P.Val<0.001]]

## Make a venn diagram
library("gplots")
vinfo <-venn(list("genes"= genes_de,"exons"= genes_w_de_exon),
    names = c("genes","exons"),show.plot =FALSE)
plot(vinfo)+
    title("Genes/exons with DE signal")
                    


**Figure 21.**
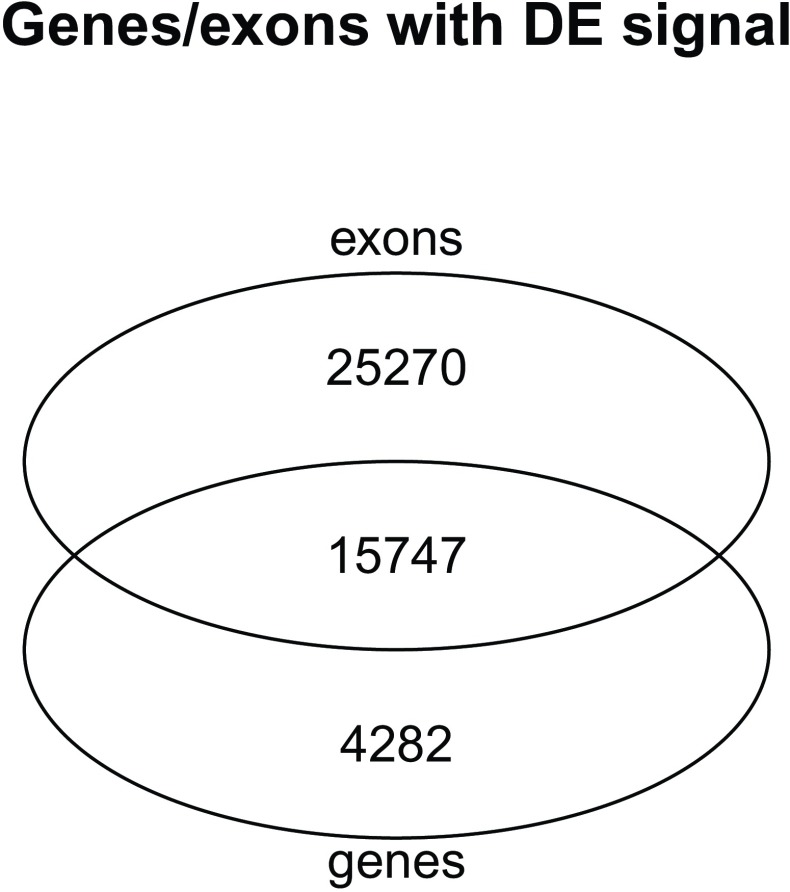
Venn diagram of the overlap between DE genes and genes with at least one exon DE.

Not all differentially expressed genes have differentially expressed exons. Moreover, genes with at least one differentially expressed exon are not necessarily differentially expressed ( Figure 21). This is in line with what was described in Figure 2B of Soneson *et al.*, 2015 ^22^.

**Figure 22.**
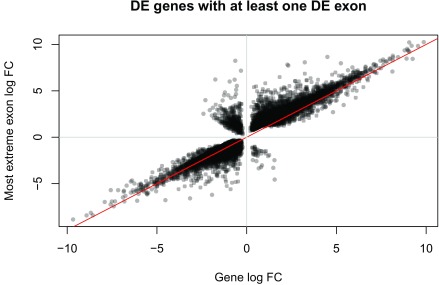
Log fold change (FC) for DE genes compared against the most extreme exon log FC among exons that are DE for the given gene.



This was just a quick example of how we can perform DE analyses at the gene and exon feature levels. We envision that more involved pipelines could be developed that leverage both feature levels, such as in Jaffe et al., 2017 ^23^. For instance, we could focus on the differentially expressed genes with at least one differentially expressed exon, and compare the direction of the DE signal versus the gene level signal as shown in Figure 22.

## Keep only the DE exons that are from a gene that is also DE
top_exon_de <- top_exon[top_exon$adj.P.Val<0.001&
	top_exon$ID%in%attr(vinfo,"intersections")[["genes:exons"]], ]

## Find the fold change that is the most extreme among the DE exons of a gene
exon_max_fc <-tapply(top_exon_de$logFC, top_exon_de$ID,function(x) {
	x[which.max(abs(x))] })

## Keep only the DE genes that match the previous selection
top_gene_de <- top[match(names(exon_max_fc),rownames(top)), ]

## Make the plot
plot(top_gene_de$logFC, exon_max_fc,pch =20,col = adjustcolor("black",1/5),
    ylab ="Most extreme exon log FC",
    xlab ="Gene log FC",
    main = "DE genes with at least one DE exon")
abline(a =0,b =1,col ="red")
abline(h =0,col ="grey80")
abline(v =0,col ="grey80")
                    


The fold change for most exons shown in Figure 22 agrees with the gene level fold change. However, some of them have opposite directions and could be interesting to study further.

### Base-pair resolution

recount2 provides bigWig coverage files (unscaled) for all samples, as well as a mean bigWig coverage file per project where each sample was scaled to 40 million 100 base-pair reads. The mean bigWig files are exactly what is needed to start an *expressed regions* analysis with derfinder ^8^. recount provides two related functions: expressed_regions() which is used to define a set of regions based on the mean bigWig file for a given project, and coverage_matrix() which based on a set of regions builds a count coverage matrix in a *RangedSummarizedExperiment* object just like the ones that are provided for genes and exons. Both functions ultimately use import.bw() from rtracklayer ^24^ which currently is not supported on Windows machines. While this presents a portability disadvantage, on the other side it allows reading portions of bigWig files from the web without having to fully download them. download_study() with type = "mean" or type = "samples" can be used to download the bigWig files, which we recommend doing when working with them extensively.

For illustrative purposes, we will use the data from chromosome 21 for the SRP045638 project. First, we obtain the expressed regions using a relatively high mean cutoff of 5. We then filter the regions to keep only the ones longer than 100 base-pairs to shorten the time needed for running coverage_matrix().



## Define expressed regions for study SRP045638, only for chromosome 21
regions <-expressed_regions("SRP045638","chr21",cutoff =5L,
    maxClusterGap =3000L)
                    




## 2017-07-30 10:39:06 loadCoverage: loading bigWig file
## http://duffel.rail.bio/recount/SRP045638/bw/mean_SRP045638.bw

## 2017-07-30 10:39:16 loadCoverage: applying the cutoff to the merged data

## 2017-07-30 10:39:16 filterData: originally there were 46709983 rows,
## now there are 46709983 rows. Meaning that 0 percent was filtered.

## 2017-07-30 10:39:16 findRegions: identifying potential segments

## 2017-07-30 10:39:16 findRegions: segmenting information

## 2017-07-30 10:39:16 .getSegmentsRle: segmenting with cutoff(s) 5

## 2017-07-30 10:39:17 findRegions: identifying candidate regions

## 2017-07-30 10:39:17 findRegions: identifying region clusters
                    




## Explore the resulting expressed regions
regions
                    




## GRanges object with 3853 ranges and 6 metadata columns:
## 	  seqnames 		 ranges strand | 	    value
## 	     <Rle> 	      <IRanges>  <Rle> | 	<numeric>
## 	1    chr21   [5026549, 5026630]      * | 6.48181250037217
## 	2    chr21   [5027935, 5027961]      * | 6.19690331706294
## 	3    chr21   [5028108, 5028225]      * | 8.99329216197386
## 	4    chr21   [5032053, 5032117]      * | 7.06828071887676
## 	5    chr21   [5032148, 5032217]      * | 6.48832686969212
##    ...      ... 		    ...    ... . 	      ...
##   3849    chr21 [46695774, 46695774]      * |  5.0290150642395
##   3850    chr21 [46695784, 46695843]      * | 5.38047295411428
##   3851    chr21 [46695865, 46695869]      * |  5.1128270149231
##   3852    chr21 [46696463, 46696486]      * | 5.25689166784286
##   3853    chr21 [46696508, 46696534]      * | 5.22988386507387
## 		      area indexStart  indexEnd cluster clusterL
## 		 <numeric>  <integer> <integer>   <Rle>    <Rle>
##  	1 531.508625030518    5026549   5026630       1     1677
## 	2 167.316389560699    5027935   5027961       1     1677
## 	3 1061.20847511292    5028108   5028225       1     1677
## 	4  459.43824672699    5032053   5032117       2     8283
## 	5 454.182880878448    5032148   5032217       2     8283
##    ... 	       ... 	  ... 	    ...     ...      ...
##   3849  5.0290150642395   46695774  46695774     708     5708
##   3850 322.828377246857   46695784  46695843     708     5708
##   3851 25.5641350746155   46695865  46695869     708     5708
##   3852 126.165400028229   46696463  46696486     708     5708
##   3853 141.206864356995   46696508  46696534     708     5708
##   -------
##   seqinfo: 1 sequence from an unspecified genome
                    




summary(width(regions))
                    




## Min. 1st Qu. Median  Mean 3rd Qu.    Max.
##  1.0     6.0   68.0 186.2   151.0 11709.0
                    




table(width(regions)>=100)
                    




##
## FALSE TRUE
## 2284 1569
                    




## Keep only the ones that are at least 100 bp long
regions <- regions[width(regions)>=100]
length(regions)
                    




## [1] 1569
                    


Now that we have a set of regions to work with, we proceed to build a *RangedSummarizedExperiment* object with the coverage counts, add the expanded metadata we built for the gene level, and scale the counts. Note that coverage_matrix() scales the base-pair coverage counts by default, which we turn off in order to use use scale_counts().



## Compute coverage matrix for study SRP045638, only for chromosome 21
## Takes about 4 minutes
rse_er <-coverage_matrix("SRP045638","chr21", regions,chunksize =2000,
    verboseLoad =FALSE,scale =FALSE)
                    




## 2017-07-30 10:39:19 railMatrix: processing regions 1 to 1569
                    




## Use the expanded metadata we built for the gene model
colData(rse_er) <-colData(rse_gene_scaled)

## Scale the coverage matrix
rse_er_scaled <-scale_counts(rse_er)

## To highlight that we scaled the counts
rm(rse_er)
                    


**Figure 23.**
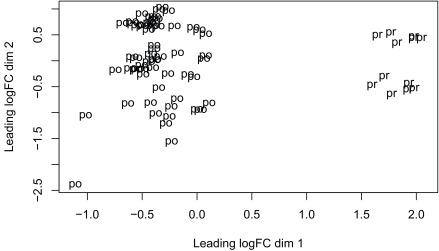
Multi-dimensional scaling plot of the expressed regions level data by age group.

**Figure 24.**
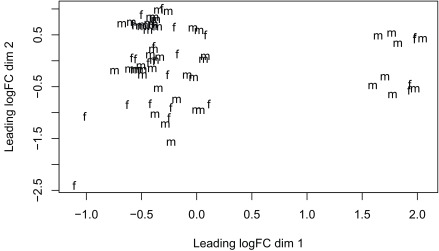
Multi-dimensional scaling plot of the expressed regions level data by sex.

**Figure 25.**
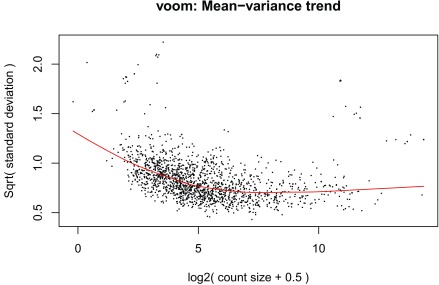
voom mean-variance plot of the expressed regions level data.

**Figure 26.**
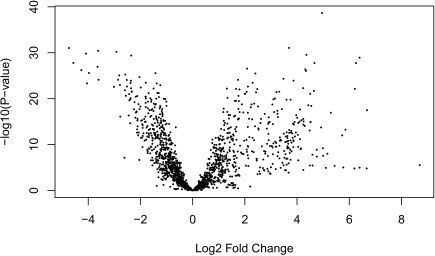
Volcano plot of the expressed regions level data. Testing for prenatal and postnatal DE adjusting for sex and RIN.



Now that we have a scaled count matrix for the expressed regions, we can proceed with the DE analysis just like we did at the gene and exon feature levels ( Figure 23, Figure 24, Figure 25, and Figure 26).

## Build DGEList object
dge_er <-DGEList(counts = assays(rse_er_scaled)$counts)

## Calculate normalization factors
dge_er <-calcNormFactors(dge_er)

## Explore the data
plotMDS(dge_er,labels = substr(colData(rse_er_scaled)$prenatal,1,2) )
                    




plotMDS(dge_er,labels = substr(colData(rse_er_scaled)$sex,1,1) )
                    




## Run voom
v_er <-voom(dge_er, design,plot =TRUE)
                    




## Run remaining parts of the DE analysis
fit_er <-lmFit(v_er, design)
fit_er <-eBayes(fit_er)
                    




## Visually explore the results
limma::volcanoplot(fit_er,coef =4)




## Number of DERs
top_er <-topTable(fit_er,number =Inf,sort.by ="none",
    coef ="prenatalpostnatal")
table(top_er$adj.P.Val<0.001)
                    




##
## FALSE TRUE
## 609 960
                    


Having identified the differentially expressed regions (DERs), we can sort all regions by their adjusted p-value.



## Sort regions by q-value
regions_by_padj <- regions[order(top_er$adj.P.Val,decreasing =FALSE)]

## Look at the top 10
regions_by_padj[1:10]
                    




## GRanges object with 10 ranges and 6 metadata columns:

##        seqnames               ranges strand |            value

##           <Rle>            <IRanges>  <Rle> |        <numeric>

##   2998    chr21 [44441692, 44442678]      * | 34.7397774041243

##   2144    chr21 [38822674, 38824916]      * | 85.5637880753472

##   3033    chr21 [44458772, 44459070]      * | 8.44090369872026

##   3029    chr21 [44458526, 44458644]      * | 5.80783885667304

##   3505    chr21 [46250498, 46250780]      * | 5.68433203882548

##   3045    chr21 [44461331, 44461480]      * | 5.82021920522054

##   1356    chr21 [33070821, 33072413]      * | 190.209820540836

##   1714    chr21 [36225565, 36225667]      * | 11.5645264560737

##   3773    chr21 [46598568, 46599629]      * | 301.859495409015

##   2254    chr21 [39928983, 39929390]      * | 233.013994795435

##                    area indexStart  indexEnd cluster clusterL

##               <numeric>  <integer> <integer>   <Rle>    <Rle>

##   2998 34288.1602978706   44441692  44442678     607    14072

##   2144 191919.576653004   38822674  38824916     435    14882

##   3033 2523.83020591736   44458772  44459070     608     4968

##   3029 691.132823944092   44458526  44458644     608     4968

##   3505 1608.66596698761   46250498  46250780     678    30649

##   3045 873.032880783081   44461331  44461480     608     4968

##   1356 303004.244121552   33070821  33072413     292     2261

##   1714 1191.14622497559   36225565  36225667     375     9845

##   3773 320574.784124374   46598568  46599629     704     6544

##   2254 95069.7098765373   39928983  39929390     464     3344

##   -------

##   seqinfo: 1 sequence from an unspecified genome





width(regions_by_padj[1:10])
                    




##  [1]  987  2243  299  119  283  150  1593  103  1062  408
                    


### Visualize regions

Since the DERs do not necessarily match the annotation, it is important to visualize them. The code for visualizing DERs can easily be adapted to visualize other regions. Although, the width and number of the regions will influence the computing resources needed to make the plots.

Because the unscaled bigWig files are available in recount2, several visualization packages can be used such as epivizr ^25^, wiggleplotr ^26^ and derfinderPlot ^8^. With all of them it is important to remember to scale the data except when visualizing the mean bigWig file for a given project.

First, we need to get the list of URLs for the bigWig files. We can either manually construct them or search them inside the recount_url table.



## Construct the list of bigWig URLs
## They have the following form:
## http://duffel.rail.bio/recount/
## project id
## /bw/
## sample run id
## .bw
bws <-paste0("http://duffel.rail.bio/recount/SRP045638/bw/",
	colData(rse_er_scaled)$bigwig_file)

## Note that they are also present in the recount_url data.frame
bws <- recount_url$url[match(colData(rse_er_scaled)$bigwig_file,
    recount_url$file_name)]

## Use the sample run IDs as the sample names
names(bws) <-colData(rse_er_scaled)$run
                    


We visualize the DERs using derfinderPlot, similar to what was done in the original publication ^13^. However, we first add a little padding to the regions: 100 base-pairs on each side.



## Add 100 bp padding on each side
regions_resized <-resize(regions_by_padj[1:10],
    width(regions_by_padj[1:10])+200,fix ="center")
                    


Next, we obtain the base-pair coverage data for each DER and scale the data to a library size of 40 million 100 base-pair reads, using the coverage AUC information we have in the metadata.



## Get the bp coverage data for the plots
library("derfinder")
regionCov <-getRegionCoverage(regions =regions_resized,files =bws,
    targetSize =40*1e6*100,totalMapped = colData(rse_er_scaled)$auc,
    verbose =FALSE)
                    


The function plotRegionCoverage() requires several pieces of annotation information for the plots that use a TxDb object. For recount2 we used Gencode v25 hg38’s annotation, which means that we need to process it manually instead of using a pre-computed TxDb package.

To create a TxDb object for Gencode v25, first we need to import the data. Since we are working only with chromosome 21 for this example, we can subset it. Next we need to add the relevant chromosome information. Some of the annotation functions we use can handle Entrez or Ensembl IDs, but not Gencode IDs. So we will make sure that we are working with Ensembl IDs before finally creating the Gencode v25 TxDb object.



## Import the Gencode v25 hg38 gene annotation
library("rtracklayer")
gencode_v25_hg38 <-import(paste0(
    "ftp://ftp.sanger.ac.uk/pub/gencode/Gencode_human/release_25/",
    "gencode.v25.annotation.gtf.gz"))


## Keep only the chr21 info
gencode_v25_hg38 <-keepSeqlevels(gencode_v25_hg38,"chr21",
	pruning.mode="coarse")

## Get the chromosome information for hg38
library("GenomicFeatures")
chrInfo <-getChromInfoFromUCSC("hg38")
                    




## Download and preprocess the ’chrominfo’ data frame ...

## OK
                    




chrInfo$chrom <-as.character(chrInfo$chrom)
chrInfo <-chrInfo[chrInfo$chrom %in%seqlevels(regions), ]
chrInfo$isCircular <-FALSE

## Assign the chromosome information to the object we will use to
## create the txdb object
si <-with(chrInfo,Seqinfo(as.character(chrom), length, isCircular,
	genome ="hg38"))
seqinfo(gencode_v25_hg38) <- si

## Switch from Gencode gene IDs to Ensembl gene IDs
gencode_v25_hg38$gene_id <-gsub("\\..*","",gencode_v25_hg38$gene_id)

## Create the TxDb object
gencode_v25_hg38_txdb <-makeTxDbFromGRanges(gencode_v25_hg38)

## Explore the TxDb object
gencode_v25_hg38_txdb
                    




## TxDb object:
## # Db type: TxDb
## # Supporting package: GenomicFeatures
## # Genome: hg38
## # transcript_nrow: 2413
## # exon_nrow: 7670
## # cds_nrow: 2623
## # Db created by: GenomicFeatures package from Bioconductor
## # Creation time: 2017-07-30 10:50:06 -0400 (Sun, 30 Jul 2017)
## # GenomicFeatures version at creation time: 1.29.8
## # RSQLite version at creation time: 2.0
## # DBSCHEMAVERSION: 1.1
                    


Now that we have a TxDb object for Gencode v25 on hg38 coordinates, we can use bumphunter’s ^27^ annotation functions for annotating the original 10 regions we were working with. Since we are using Ensembl instead of Entrez gene IDs, we need to pass this information to annotateTranscripts(). Otherwise, the function will fail to retrieve the gene symbols.



library("bumphunter")
## Annotate all transcripts for gencode v25 based on the TxDb object
## we built previously.
ann_gencode_v25_hg38 <-annotateTranscripts(gencode_v25_hg38_txdb,
    annotationPackage ="org.Hs.eg.db",
    mappingInfo = list("column"="ENTREZID","keytype"="ENSEMBL",
    "multiVals"="first"))
                    




## Getting TSS and TSE.

## Getting CSS and CSE.

## Getting exons.

## Annotating genes.

## ’select()’ returned 1:many mapping between keys and columns
                    




## Annotate the regions of interest
## Note that we are using the original regions, not the resized ones
nearest_ann <-matchGenes(regions_by_padj[1:10], ann_gencode_v25_hg38)
                    


The final piece we need to run plotRegionCoverage() is information about which base-pairs are exonic, intronic, etc. This is done via the annotateRegions() function in derfinder, which itself requires prior processing of the TxDb information by makeGenomicState().



## Create the genomic state object using the gencode TxDb object
gs_gencode_v25_hg38 <-makeGenomicState(gencode_v25_hg38_txdb,
	chrs = seqlevels(regions))
                    




## ’select()’ returned 1:1 mapping between keys and columns
                    




## Annotate the original regions
regions_ann <-annotateRegions(regions_resized,
	gs_gencode_v25_hg38$fullGenome)
                    




## 2017-07-30 10:50:35 annotateRegions: counting

## 2017-07-30 10:50:35 annotateRegions: annotating
                    


We can finally use plotRegionCoverage() to visualize the top 10 regions coloring by whether they are prenatal or postnatal samples. Known exons are shown in dark blue, introns in light blue.



library("derfinderPlot")
plotRegionCoverage(regions =regions_resized,regionCoverage =regionCov,
    groupInfo = colData(rse_er_scaled)$prenatal,
    nearestAnnotation =nearest_ann,
    annotatedRegions =regions_ann,
    txdb =gencode_v25_hg38_txdb,
    scalefac =1,ylab ="Coverage (RP40M, 100bp)",
    ask =FALSE,verbose =FALSE)
                    


**Figure 27.**
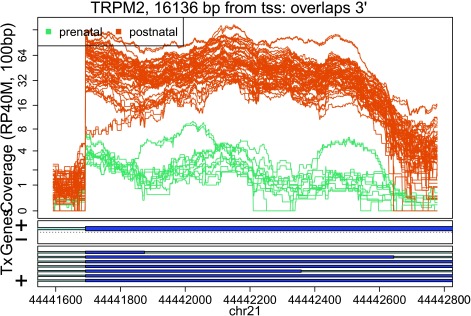
Base-pair resolution plot of differentially expressed region 1.

**Figure 28.**
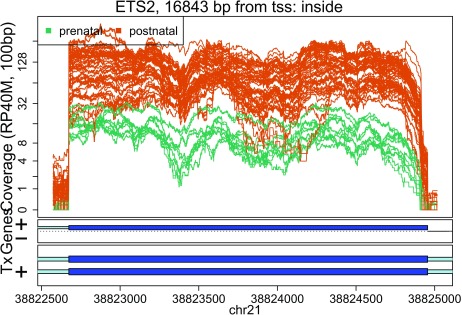
Base-pair resolution plot of differentially expressed region 2.

**Figure 29.**
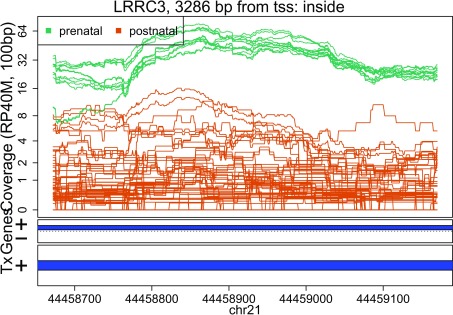
Base-pair resolution plot of differentially expressed region 3.

In these plots we can see that some DERs match known exons ( Figure 28, Figure 34, Figure 36), some are longer than known exons ( Figure 27, Figure 33, Figure 35), and others are exon fragments ( Figure 29– Figure 32) which could be due to the cutoff used. Note that Figure 33 could be shorter than a known exon due to a coverage dip.

**Figure 30.**
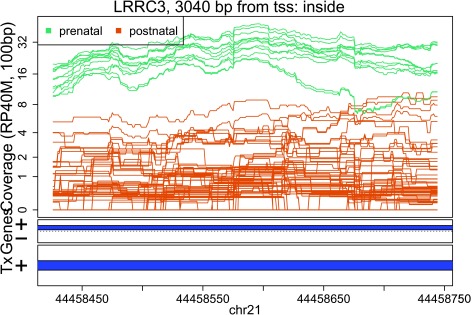
Base-pair resolution plot of differentially expressed region 4.

**Figure 31.**
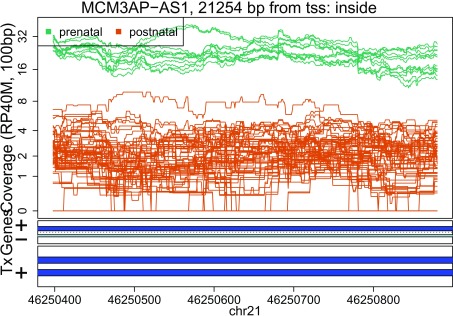
Base-pair resolution plot of differentially expressed region 5.

**Figure 32.**
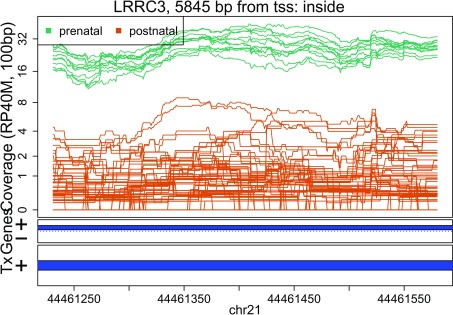
Base-pair resolution plot of differentially expressed region 6.

**Figure 33.**
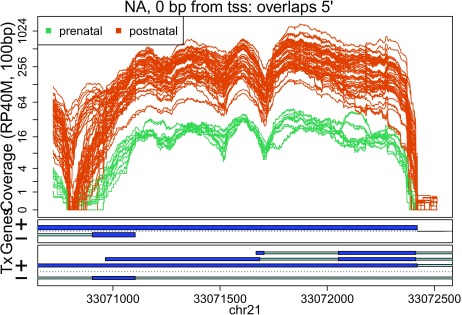
Base-pair resolution plot of differentially expressed region 7.

**Figure 34.**
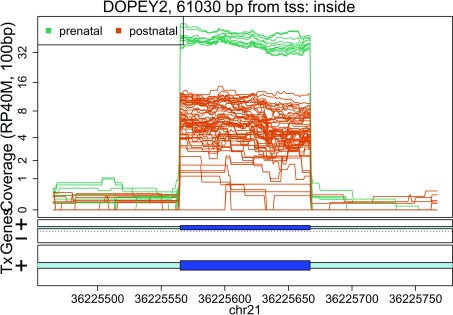
Base-pair resolution plot of differentially expressed region 8.

**Figure 35.**
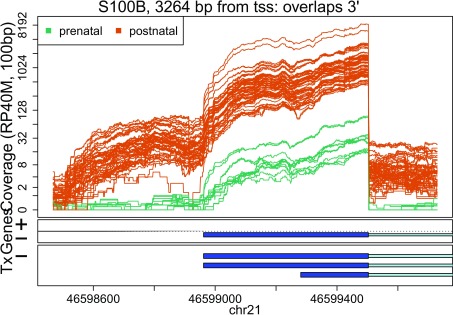
Base-pair resolution plot of differentially expressed region 9.

**Figure 36.**
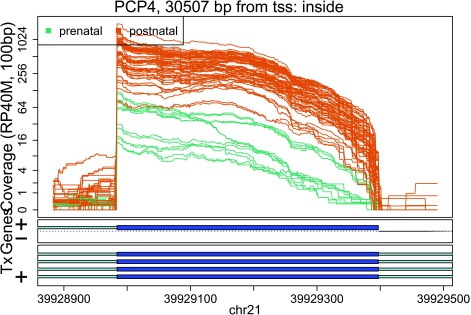
Base-pair resolution plot of differentially expressed region 10.

## Summary

In this workflow we described in detail the available data in recount2, how the coverage count matrices were computed, the metadata included in recount2 and how to get new phenotypic information from other sources. We showed how to perform a DE analysis at the gene and exon levels as well as use an annotation-agnostic approach. Finally, we explained how to visualize the base-pair information for a given set of regions. This workflow constitutes a strong basis to leverage the recount2 data for human RNA-seq analyses.

## Supplementary material

**Supplementary File 1: A pre-computed version of the differential expression report.**

**Supplementary File 2: Session information.** This workflow was created using BiocWorkflowTools ^28^.

The session information is available in this file. The most recent version of this workflow is available via Bioconductor at http://bioconductor.org/help/workflows/.
