# Supplementary material for: recount workflow: Accessing over 70,000 human RNA-seq samples with Bioconductor
Source: F1000Res. 2017 Aug 24;6:1558. [Version 1] doi: 10.12688/f1000research.12223.1 (PMC5621122; doi:10.12688/f1000research.12223.1)
Supplement: Supplementary file 1 [file f1000research-6-13233-s0000.tgz › 59ee59e9-6279-4c31-9305-5d982fdd8677.html]

Code 

- Show All Code
- Hide All Code

# 1 Basic limma results exploration

Project: SRP045638 gene results with limma-voom.

# 2 Introduction

This report is meant to help explore limma (Ritchie, Phipson, Wu, Hu, et al., 2015) results and was generated using the `regionReport` (Collado-Torres, Jaffe, and Leek, 2016) package. While the report is rich, it is meant to just start the exploration of the results and exemplify some of the code used to do so. If you need a more in-depth analysis for your specific data set you might want to use the `customCode` argument. This report is based on the vignette of the `DESeq2` (Love, Huber, and Anders, 2014) package which you can find here.

## 2.1 Code setup

This section contains the code for setting up the rest of the report.

```
## knitrBoostrap and device chunk options
load_install('knitr')
opts_chunk$set(bootstrap.show.code = FALSE, dev = device)
if(!outputIsHTML) opts_chunk$set(bootstrap.show.code = FALSE, dev = device, echo = FALSE)
```

```
#### Libraries needed

## Bioconductor
load_install('DESeq2')
if(isEdgeR) load_install('edgeR')

## CRAN
load_install('ggplot2')
if(!is.null(theme)) theme_set(theme)
load_install('knitr')
if(is.null(colors)) {
    load_install('RColorBrewer')
}
load_install('pheatmap')
load_install('DT')
load_install('devtools')

## Working behind the scenes
# load_install('knitcitations')
# load_install('rmarkdown')
## Optionally
# load_install('knitrBootstrap')

#### Code setup

## For ggplot
res.df <- as.data.frame(res)

## Sort results by adjusted p-values
ord <- order(res.df$padj, decreasing = FALSE)
res.df <- res.df[ord, ]
res.df <- cbind(data.frame(Feature = rownames(res.df)), res.df)
rownames(res.df) <- NULL
```

# 3 PCA

```
## Transform count data
rld <- tryCatch(rlog(dds), error = function(e) { rlog(dds, fitType = 'mean') })

## Perform PCA analysis and make plot
plotPCA(rld, intgroup = intgroup)
```

```
## Get percent of variance explained
data_pca <- plotPCA(rld, intgroup = intgroup, returnData = TRUE)
percentVar <- round(100 * attr(data_pca, "percentVar"))
```

The above plot shows the first two principal components that explain the variability in the data using the regularized log count data. If you are unfamiliar with principal component analysis, you might want to check the Wikipedia entry or this interactive explanation. In this case, the first and second principal component explain 50 and 13 percent of the variance respectively.

# 4 Sample-to-sample distances

```
## Obtain the sample euclidean distances
sampleDists <- dist(t(assay(rld)))
sampleDistMatrix <- as.matrix(sampleDists)
## Add names based on intgroup
rownames(sampleDistMatrix) <- apply(as.data.frame(colData(rld)[, intgroup]), 1,
    paste, collapse = ' : ')
colnames(sampleDistMatrix) <- NULL

## Define colors to use for the heatmap if none were supplied
if(is.null(colors)) {
    colors <- colorRampPalette( rev(brewer.pal(9, "Blues")) )(255)
}

## Make the heatmap
pheatmap(sampleDistMatrix, clustering_distance_rows = sampleDists,
    clustering_distance_cols = sampleDists, color = colors)
```

This plot shows how samples are clustered based on their euclidean distance using the regularized log transformed count data. This figure gives an overview of how the samples are hierarchically clustered. It is a complementary figure to the PCA plot.

# 5 MA plots

This section contains three MA plots (see Wikipedia) that compare the mean of the normalized counts against the log fold change. They show one point per feature. The points are shown in red if the feature has an adjusted p-value less than `alpha`, that is, the statistically significant features are shown in red.

```
## MA plot with alpha used in DESeq2::results()
plotMA(res, alpha = metadata(res)$alpha, main = paste('MA plot with alpha =',
    metadata(res)$alpha))
```

This first plot shows uses `alpha` = 0.001, which is the `alpha` value used to determine which resulting features were significant when running the function `DESeq2::results()`.

```
## MA plot with alpha = 1/2 of the alpha used in DESeq2::results()
plotMA(res, alpha = metadata(res)$alpha / 2,
    main = paste('MA plot with alpha =', metadata(res)$alpha / 2))
```

This second MA plot uses `alpha` = 510^{-4} and can be used agains the first MA plot to identify which features have adjusted p-values between 510^{-4} and 0.001.

```
## MA plot with alpha corresponding to the one that gives the nBest features
nBest.actual <- min(nBest, nrow(head(res.df, n = nBest)))
nBest.alpha <- head(res.df, n = nBest)$padj[nBest.actual]
plotMA(res, alpha = nBest.alpha * 1.00000000000001,
    main = paste('MA plot for top', nBest.actual, 'features'))
```

The third and final MA plot uses an alpha such that the top 500 features are shown in the plot. These are the features that whose details are included in the *top features* interactive table.

# 6 P-values distribution

```
## P-value histogram plot
ggplot(res.df[!is.na(res.df$pvalue), ], aes(x = pvalue)) +
    geom_histogram(alpha=.5, position='identity', bins = 50) +
    labs(title='Histogram of unadjusted p-values') +
    xlab('Unadjusted p-values') +
    xlim(c(0, 1.0005))
```

This plot shows a histogram of the unadjusted p-values. It might be skewed right or left, or flat as shown in the Wikipedia examples. The shape depends on the percent of features that are differentially expressed. For further information on how to interpret a histogram of p-values check David Robinson’s post on this topic.

```
## P-value distribution summary
summary(res.df$pvalue)
```

```
##      Min.   1st Qu.    Median      Mean   3rd Qu.      Max. 
## 0.0000000 0.0000000 0.0001971 0.1144422 0.0787971 0.9998200
```

This is the numerical summary of the distribution of the p-values.

```
## Split features by different p-value cutoffs
pval_table <- lapply(c(1e-04, 0.001, 0.01, 0.025, 0.05, 0.1, 0.2, 0.3, 0.4, 0.5,
    0.6, 0.7, 0.8, 0.9, 1), function(x) {
    data.frame('Cut' = x, 'Count' = sum(res.df$pvalue <= x, na.rm = TRUE))
})
pval_table <- do.call(rbind, pval_table)
if(outputIsHTML) {
    kable(pval_table, format = 'markdown', align = c('c', 'c'))
} else {
    kable(pval_table)
}
```

| Cut | Count |
| --- | --- |
| 0.0001 | 18153 |
| 0.0010 | 20824 |
| 0.0100 | 24201 |
| 0.0250 | 25835 |
| 0.0500 | 27274 |
| 0.1000 | 28930 |
| 0.2000 | 30802 |
| 0.3000 | 32163 |
| 0.4000 | 33244 |
| 0.5000 | 34167 |
| 0.6000 | 34956 |
| 0.7000 | 35710 |
| 0.8000 | 36424 |
| 0.9000 | 37137 |
| 1.0000 | 37817 |

This table shows the number of features with p-values less or equal than some commonly used cutoff values.

# 7 Adjusted p-values distribution

```
## Adjusted p-values histogram plot
ggplot(res.df[!is.na(res.df$padj), ], aes(x = padj)) +
    geom_histogram(alpha=.5, position='identity', bins = 50) +
    labs(title=paste('Histogram of', elementMetadata(res)$description[grep('adjusted', elementMetadata(res)$description)])) +
    xlab('Adjusted p-values') +
    xlim(c(0, 1.0005))
```

This plot shows a histogram of the . It might be skewed right or left, or flat as shown in the Wikipedia examples.

```
## Adjusted p-values distribution summary
summary(res.df$padj)
```

```
##      Min.   1st Qu.    Median      Mean   3rd Qu.      Max. 
## 0.0000000 0.0000000 0.0003943 0.1265450 0.1050618 0.9998200
```

This is the numerical summary of the distribution of the .

```
## Split features by different adjusted p-value cutoffs
padj_table <- lapply(c(1e-04, 0.001, 0.01, 0.025, 0.05, 0.1, 0.2, 0.3, 0.4, 0.5,
    0.6, 0.7, 0.8, 0.9, 1), function(x) {
    data.frame('Cut' = x, 'Count' = sum(res.df$padj <= x, na.rm = TRUE))
})
padj_table <- do.call(rbind, padj_table)
if(outputIsHTML) {
    kable(padj_table, format = 'markdown', align = c('c', 'c'))
} else {
    kable(padj_table)
}
```

| Cut | Count |
| --- | --- |
| 0.0001 | 17372 |
| 0.0010 | 20029 |
| 0.0100 | 23427 |
| 0.0250 | 25113 |
| 0.0500 | 26500 |
| 0.1000 | 28217 |
| 0.2000 | 30164 |
| 0.3000 | 31521 |
| 0.4000 | 32679 |
| 0.5000 | 33675 |
| 0.6000 | 34549 |
| 0.7000 | 35374 |
| 0.8000 | 36156 |
| 0.9000 | 36975 |
| 1.0000 | 37817 |

This table shows the number of features with less or equal than some commonly used cutoff values.

# 8 Top features

This interactive table shows the top 500 features ordered by their . Use the search function to find your feature of interest or sort by one of the columns.

```
## Add search url if appropriate
if(!is.null(searchURL) & outputIsHTML) {
    res.df$Feature <- paste0('<a href="', searchURL, res.df$Feature, '">',
        res.df$Feature, '</a>')
}

for(i in which(colnames(res.df) %in% c('pvalue', 'padj'))) res.df[, i] <- format(res.df[, i], scientific = TRUE)

if(outputIsHTML) {
    datatable(head(res.df, n = nBest), options = list(pagingType='full_numbers', pageLength=10, scrollX='100%'), escape = FALSE, rownames = FALSE) %>% formatRound(which(!colnames(res.df) %in% c('pvalue', 'padj', 'Feature')), digits)
} else {
    res.df_top <- head(res.df, n = 20)
    for(i in which(!colnames(res.df) %in% c('pvalue', 'padj', 'Feature'))) res.df_top[, i] <- round(res.df_top[, i], digits)
    kable(res.df_top)
}
```

# 9 Count plots top features

This section contains plots showing the normalized counts per sample for each group of interest. Only the best 20 features are shown, ranked by their . The Y axis is on the log10 scale and the feature name is shown in the title of each plot.

```
plotCounts_gg <- function(i, dds, intgroup) {
    group <- if (length(intgroup) == 1) {
        colData(dds)[[intgroup]]
    } else if (length(intgroup) == 2) {
        lvls <- as.vector(t(outer(levels(colData(dds)[[intgroup[1]]]), 
            levels(colData(dds)[[intgroup[2]]]), function(x, 
                y) paste(x, y, sep = " : "))))
        droplevels(factor(apply(as.data.frame(colData(dds)[, 
            intgroup, drop = FALSE]), 1, paste, collapse = " : "), 
            levels = lvls))
    } else {
        factor(apply(as.data.frame(colData(dds)[, intgroup, drop = FALSE]), 
            1, paste, collapse = " : "))
    }
    data <- plotCounts(dds, gene=i, intgroup=intgroup, returnData = TRUE)
    data <- cbind(data, data.frame('group' = group))
    main <- rownames(dds)[i]

    ggplot(data, aes(x = group, y = count)) + geom_point() + ylab('Normalized count') + ggtitle(main) + coord_trans(y = "log10")
}
for(i in head(ord, nBestFeatures)) {
    print(plotCounts_gg(i, dds = dds, intgroup = intgroup))
}
```

# 10 Reproducibility

The input for this report was generated with limma (Ritchie, Phipson, Wu, Hu, et al., 2015) and the resulting features were called significantly differentially expressed if their were less than `alpha` = 0.001. This report was generated in path /Users/lcollado/Dropbox/Code/recount-workflow using the following call to DESeq2Report():

```
## DESeq2Report(dds = dds, project = "SRP045638 gene results with limma-voom", 
##     intgroup = c("prenatal", "sex"), res = limma_res, outdir = "SRP045638", 
##     output = "gene_report", software = "limma")
```

Date the report was generated.

```
## [1] "2017-07-30 10:35:54 EDT"
```

Wallclock time spent generating the report.

```
## Time difference of 23.633 mins
```

`R` session information.

```
## Session info ----------------------------------------------------------------------------------------------------------
```

```
##  setting  value                       
##  version  R version 3.4.1 (2017-06-30)
##  system   x86_64, darwin15.6.0        
##  ui       X11                         
##  language (EN)                        
##  collate  en_US.UTF-8                 
##  tz       America/New_York            
##  date     2017-07-30
```

```
## Packages --------------------------------------------------------------------------------------------------------------
```

```
##  package                * version  date       source         
##  acepack                  1.4.1    2016-10-29 CRAN (R 3.4.0) 
##  annotate                 1.55.0   2017-05-04 Bioconductor   
##  AnnotationDbi          * 1.39.2   2017-07-26 Bioconductor   
##  AnnotationFilter         1.1.3    2017-06-28 Bioconductor   
##  AnnotationHub            2.9.5    2017-06-06 Bioconductor   
##  assertthat               0.2.0    2017-04-11 cran (@0.2.0)  
##  backports                1.1.0    2017-05-22 CRAN (R 3.4.0) 
##  base                   * 3.4.1    2017-07-17 local          
##  base64enc                0.1-3    2015-07-28 cran (@0.1-3)  
##  bibtex                   0.4.2    2017-06-30 CRAN (R 3.4.1) 
##  Biobase                * 2.37.2   2017-05-05 Bioconductor   
##  BiocGenerics           * 0.23.0   2017-05-04 Bioconductor   
##  BiocInstaller            1.27.2   2017-05-04 Bioconductor   
##  BiocParallel             1.11.4   2017-06-27 Bioconductor   
##  BiocStyle              * 2.5.8    2017-07-21 Bioconductor   
##  BiocWorkflowTools        1.3.9    2017-06-09 Bioconductor   
##  biomaRt                  2.33.3   2017-06-17 Bioconductor   
##  Biostrings               2.45.3   2017-07-21 Bioconductor   
##  biovizBase               1.25.1   2017-05-05 Bioconductor   
##  bit                      1.1-12   2014-04-09 CRAN (R 3.4.0) 
##  bit64                    0.9-7    2017-05-08 CRAN (R 3.4.0) 
##  bitops                   1.0-6    2013-08-17 cran (@1.0-6)  
##  blob                     1.1.0    2017-06-17 CRAN (R 3.4.0) 
##  bookdown                 0.4      2017-05-20 CRAN (R 3.4.0) 
##  BSgenome                 1.45.1   2017-05-05 Bioconductor   
##  bumphunter             * 1.17.2   2017-05-20 Bioconductor   
##  caTools                  1.17.1   2014-09-10 cran (@1.17.1) 
##  checkmate                1.8.3    2017-07-03 CRAN (R 3.4.1) 
##  cluster                  2.0.6    2017-03-10 CRAN (R 3.4.1) 
##  clusterProfiler        * 3.5.5    2017-07-25 Bioconductor   
##  codetools                0.2-15   2016-10-05 CRAN (R 3.4.1) 
##  colorspace               1.3-2    2016-12-14 cran (@1.3-2)  
##  compiler                 3.4.1    2017-07-17 local          
##  curl                     2.8.1    2017-07-21 CRAN (R 3.4.1) 
##  data.table               1.10.4   2017-02-01 cran (@1.10.4) 
##  datasets               * 3.4.1    2017-07-17 local          
##  DBI                      0.7      2017-06-18 CRAN (R 3.4.0) 
##  DEFormats                1.5.0    2017-05-04 Bioconductor   
##  DelayedArray           * 0.3.19   2017-07-28 Bioconductor   
##  derfinder              * 1.11.5   2017-07-07 Bioconductor   
##  derfinderHelper          1.11.0   2017-05-04 Bioconductor   
##  derfinderPlot          * 1.11.0   2017-05-05 Bioconductor   
##  DESeq2                 * 1.17.11  2017-07-22 Bioconductor   
##  devtools               * 1.13.2   2017-06-02 CRAN (R 3.4.0) 
##  dichromat                2.0-0    2013-01-24 cran (@2.0-0)  
##  digest                   0.6.12   2017-01-27 CRAN (R 3.4.0) 
##  DO.db                    2.9      2017-05-04 Bioconductor   
##  doRNG                    1.6.6    2017-04-10 CRAN (R 3.4.0) 
##  DOSE                   * 3.3.1    2017-06-02 Bioconductor   
##  downloader               0.4      2015-07-09 CRAN (R 3.4.0) 
##  DT                     * 0.2      2016-08-09 cran (@0.2)    
##  edgeR                  * 3.19.3   2017-07-01 Bioconductor   
##  ensembldb                2.1.10   2017-06-17 Bioconductor   
##  evaluate                 0.10.1   2017-06-24 CRAN (R 3.4.1) 
##  fastmatch                1.1-0    2017-01-28 CRAN (R 3.4.0) 
##  fgsea                    1.3.1    2017-05-04 Bioconductor   
##  foreach                * 1.4.3    2015-10-13 CRAN (R 3.4.0) 
##  foreign                  0.8-69   2017-06-22 CRAN (R 3.4.1) 
##  Formula                  1.2-2    2017-07-10 CRAN (R 3.4.1) 
##  gdata                    2.18.0   2017-06-06 CRAN (R 3.4.0) 
##  genefilter               1.59.0   2017-05-04 Bioconductor   
##  geneplotter              1.55.0   2017-05-04 Bioconductor   
##  GenomeInfoDb           * 1.13.4   2017-06-06 Bioconductor   
##  GenomeInfoDbData         0.99.1   2017-07-17 Bioconductor   
##  GenomicAlignments        1.13.4   2017-07-20 Bioconductor   
##  GenomicFeatures        * 1.29.8   2017-07-20 Bioconductor   
##  GenomicFiles             1.13.10  2017-07-17 Bioconductor   
##  GenomicRanges          * 1.29.12  2017-07-28 Bioconductor   
##  GEOquery                 2.43.0   2017-05-04 Bioconductor   
##  GGally                   1.3.1    2017-06-08 CRAN (R 3.4.0) 
##  ggbio                    1.25.3   2017-07-01 Bioconductor   
##  ggplot2                * 2.2.1    2016-12-30 CRAN (R 3.4.0) 
##  git2r                    0.19.0   2017-07-19 CRAN (R 3.4.1) 
##  GO.db                    3.4.1    2017-05-04 Bioconductor   
##  GOSemSim                 2.3.1    2017-05-23 Bioconductor   
##  gplots                 * 3.0.1    2016-03-30 cran (@3.0.1)  
##  graph                    1.55.0   2017-05-04 Bioconductor   
##  graphics               * 3.4.1    2017-07-17 local          
##  grDevices              * 3.4.1    2017-07-17 local          
##  grid                     3.4.1    2017-07-17 local          
##  gridExtra                2.2.1    2016-02-29 CRAN (R 3.4.0) 
##  gtable                   0.2.0    2016-02-26 CRAN (R 3.4.0) 
##  gtools                   3.5.0    2015-05-29 cran (@3.5.0)  
##  highr                    0.6      2016-05-09 cran (@0.6)    
##  Hmisc                    4.0-3    2017-05-02 CRAN (R 3.4.0) 
##  htmlTable                1.9      2017-01-26 CRAN (R 3.4.0) 
##  htmltools                0.3.6    2017-04-28 CRAN (R 3.4.0) 
##  htmlwidgets              0.9      2017-07-10 CRAN (R 3.4.1) 
##  httpuv                   1.3.5    2017-07-04 CRAN (R 3.4.1) 
##  httr                     1.2.1    2016-07-03 CRAN (R 3.4.0) 
##  igraph                   1.1.2    2017-07-21 CRAN (R 3.4.1) 
##  interactiveDisplayBase   1.15.0   2017-05-04 Bioconductor   
##  IRanges                * 2.11.12  2017-07-22 Bioconductor   
##  iterators              * 1.0.8    2015-10-13 CRAN (R 3.4.0) 
##  jsonlite                 1.5      2017-06-01 CRAN (R 3.4.0) 
##  KernSmooth               2.23-15  2015-06-29 CRAN (R 3.4.1) 
##  knitcitations            1.0.8    2017-07-04 CRAN (R 3.4.1) 
##  knitr                  * 1.16     2017-05-18 CRAN (R 3.4.0) 
##  knitrBootstrap           1.0.1    2017-07-19 CRAN (R 3.4.1) 
##  labeling                 0.3      2014-08-23 cran (@0.3)    
##  lattice                  0.20-35  2017-03-25 CRAN (R 3.4.1) 
##  latticeExtra             0.6-28   2016-02-09 CRAN (R 3.4.0) 
##  lazyeval                 0.2.0    2016-06-12 cran (@0.2.0)  
##  limma                  * 3.33.6   2017-07-26 Bioconductor   
##  locfit                 * 1.5-9.1  2013-04-20 CRAN (R 3.4.0) 
##  lubridate                1.6.0    2016-09-13 CRAN (R 3.4.0) 
##  magrittr                 1.5      2014-11-22 cran (@1.5)    
##  markdown                 0.8      2017-04-20 cran (@0.8)    
##  Matrix                   1.2-10   2017-05-03 CRAN (R 3.4.1) 
##  matrixStats            * 0.52.2   2017-04-14 CRAN (R 3.4.0) 
##  memoise                  1.1.0    2017-04-21 CRAN (R 3.4.0) 
##  methods                * 3.4.1    2017-07-17 local          
##  mime                     0.5      2016-07-07 CRAN (R 3.4.0) 
##  munsell                  0.4.3    2016-02-13 cran (@0.4.3)  
##  nnet                     7.3-12   2016-02-02 CRAN (R 3.4.1) 
##  org.Hs.eg.db           * 3.4.1    2017-05-04 Bioconductor   
##  OrganismDbi              1.19.0   2017-05-05 Bioconductor   
##  parallel               * 3.4.1    2017-07-17 local          
##  pheatmap               * 1.0.8    2015-12-11 CRAN (R 3.4.0) 
##  pkgconfig                2.0.1    2017-03-21 CRAN (R 3.4.0) 
##  pkgmaker                 0.22     2014-05-14 CRAN (R 3.4.0) 
##  plyr                     1.8.4    2016-06-08 cran (@1.8.4)  
##  prettyunits              1.0.2    2015-07-13 CRAN (R 3.4.0) 
##  progress                 1.1.2    2016-12-14 CRAN (R 3.4.0) 
##  ProtGenerics             1.9.0    2017-05-04 Bioconductor   
##  qvalue                   2.9.0    2017-05-04 Bioconductor   
##  R6                       2.2.2    2017-06-17 CRAN (R 3.4.0) 
##  RBGL                     1.53.0   2017-05-04 Bioconductor   
##  RColorBrewer           * 1.1-2    2014-12-07 cran (@1.1-2)  
##  Rcpp                     0.12.12  2017-07-15 CRAN (R 3.4.1) 
##  RCurl                    1.95-4.8 2016-03-01 cran (@1.95-4.)
##  recount                * 1.3.2    2017-07-29 Bioconductor   
##  RefManageR               0.14.12  2017-07-04 CRAN (R 3.4.1) 
##  regionReport           * 1.11.2   2017-07-15 Bioconductor   
##  registry                 0.3      2015-07-08 CRAN (R 3.4.0) 
##  rentrez                  1.1.0    2017-06-01 CRAN (R 3.4.0) 
##  reshape                  0.8.6    2016-10-21 CRAN (R 3.4.0) 
##  reshape2                 1.4.2    2016-10-22 cran (@1.4.2)  
##  rlang                    0.1.1    2017-05-18 CRAN (R 3.4.0) 
##  rmarkdown                1.6      2017-06-15 CRAN (R 3.4.0) 
##  rngtools                 1.2.4    2014-03-06 CRAN (R 3.4.0) 
##  rpart                    4.1-11   2017-03-13 CRAN (R 3.4.1) 
##  rprojroot                1.2      2017-01-16 cran (@1.2)    
##  Rsamtools                1.29.0   2017-05-05 Bioconductor   
##  RSQLite                  2.0      2017-06-19 CRAN (R 3.4.1) 
##  rtracklayer            * 1.37.3   2017-07-22 Bioconductor   
##  rvcheck                  0.0.9    2017-07-10 CRAN (R 3.4.1) 
##  S4Vectors              * 0.15.5   2017-06-27 Bioconductor   
##  scales                   0.4.1    2016-11-09 cran (@0.4.1)  
##  shiny                    1.0.3    2017-04-26 CRAN (R 3.4.0) 
##  splines                  3.4.1    2017-07-17 local          
##  stats                  * 3.4.1    2017-07-17 local          
##  stats4                 * 3.4.1    2017-07-17 local          
##  stringi                  1.1.5    2017-04-07 cran (@1.1.5)  
##  stringr                  1.2.0    2017-02-18 cran (@1.2.0)  
##  SummarizedExperiment   * 1.7.5    2017-06-22 Bioconductor   
##  survival                 2.41-3   2017-04-04 CRAN (R 3.4.1) 
##  tibble                   1.3.3    2017-05-28 CRAN (R 3.4.0) 
##  tidyr                    0.6.3    2017-05-15 CRAN (R 3.4.0) 
##  tools                    3.4.1    2017-07-17 local          
##  utils                  * 3.4.1    2017-07-17 local          
##  VariantAnnotation        1.23.6   2017-07-22 Bioconductor   
##  withr                    2.0.0    2017-07-28 CRAN (R 3.4.1) 
##  XML                      3.98-1.9 2017-06-19 CRAN (R 3.4.1) 
##  xml2                     1.1.1    2017-01-24 cran (@1.1.1)  
##  xtable                   1.8-2    2016-02-05 cran (@1.8-2)  
##  XVector                  0.17.0   2017-05-04 Bioconductor   
##  yaml                     2.1.14   2016-11-12 cran (@2.1.14) 
##  zlibbioc                 1.23.0   2017-05-04 Bioconductor
```

Pandoc version used: 1.19.2.1.

# 11 Bibliography

This report was created with `regionReport` (Collado-Torres, Jaffe, and Leek, 2016) using `rmarkdown` (Allaire, Cheng, Xie, McPherson, et al., 2017) while `knitr` (Xie, 2014) and `DT` (Xie, 2016) were running behind the scenes. `pheatmap` (Kolde, 2015) was used to create the sample distances heatmap. Several plots were made with `ggplot2` (Wickham, 2009).

Citations made with `knitcitations` (Boettiger, 2017). The BibTeX file can be found here.

[1] J. Allaire, J. Cheng, Y. Xie, J. McPherson, et al. *rmarkdown: Dynamic Documents for R*. R package version 1.6. 2017. URL: https://CRAN.R-project.org/package=rmarkdown.

[1] C. Boettiger. *knitcitations: Citations for ‘Knitr’ Markdown Files*. R package version 1.0.8. 2017. URL: https://CRAN.R-project.org/package=knitcitations.

```
## No encoding supplied: defaulting to UTF-8.
```

[1] R. Kolde. *pheatmap: Pretty Heatmaps*. R package version 1.0.8. 2015. URL: https://CRAN.R-project.org/package=pheatmap.

```
## No encoding supplied: defaulting to UTF-8.
```

[1] M. E. Ritchie, B. Phipson, D. Wu, Y. Hu, et al. “limma powers differential expression analyses for RNA-sequencing and microarray studies”. In: *Nucleic Acids Research* 43.7 (2015), p. e47.

[1] H. Wickham. *ggplot2: Elegant Graphics for Data Analysis*. Springer-Verlag New York, 2009. ISBN: 978-0-387-98140-6. URL: http://ggplot2.org.

[1] Y. Xie. *DT: A Wrapper of the JavaScript Library ‘DataTables’*. R package version 0.2. 2016. URL: https://CRAN.R-project.org/package=DT.

[1] Y. Xie. “knitr: A Comprehensive Tool for Reproducible Research in R”. In: *Implementing Reproducible Computational Research*. Ed. by V. Stodden, F. Leisch and R. D. Peng. ISBN 978-1466561595. Chapman and Hall/CRC, 2014. URL: http://www.crcpress.com/product/isbn/9781466561595.
