# Supplementary material for: recount workflow: Accessing over 70,000 human RNA-seq samples with Bioconductor
Source: F1000Res. 2017 Aug 24;6:1558. [Version 1] doi: 10.12688/f1000research.12223.1 (PMC5621122; doi:10.12688/f1000research.12223.1)
Supplement: Supplementary file 2 [file f1000research-6-13233-s0001.tgz › 484279a3-25b1-44df-9332-2e880ca66514.pdf]

recount workflow session information

Leonardo Collado-Torres*[1,2], Abhinav Nellore[3,4,5], and Andrew E. Jaffe[1,2,6,7]

[1]Lieber Institute for Brain Development, Johns Hopkins Medical Campus
[2]Center for Computational Biology, Johns Hopkins University
[3]Department of Biomedical Engineering, Oregon Health and Science University
[4]Department of Surgery, Oregon Health and Science University
[5]Computational Biology Program, Oregon Health and Science University
[6]Department of Biostatistics, Johns Hopkins Bloomberg School of Public Health
[7]Department of Mental Health, Johns Hopkins Bloomberg School of Public Health

```r
## Final list of files created
dir("SRP045638")
```

```
## [1] "gene_report.bib"          "gene_report.html"
## [3] "rse_exon.Rdata"           "rse_gene.Rdata"
## [5] "SraRunTable.txt"          "SupplementaryFile1.html"
```

```r
## Pandoc information
library("rmarkdown")
```

```
##
## Attaching package: 'rmarkdown'

## The following objects are masked from 'package:BiocStyle':
##
##     html_document, md_document, pdf_document
```

```r
pandoc_version()
```

```
## [1] '1.19.2.1'
```

```r
## Time for reproducing this workflow, in minutes
round(proc.time()[3] / 60, 1)
```

```
## elapsed
##    39.8
```

```r
options(width = 100)
library("devtools")
session_info()
```

```
## Session info -----------------------------------------------------------------------------------

##  setting  value
##  version  R version 3.4.1 (2017-06-30)
##  system   x86_64, darwin15.6.0
##  ui       X11
##  language (EN)
##  collate  en_US.UTF-8
##  tz       America/New_York
##  date     2017-07-30

## Packages ---------------------------------------------------------------------------------------
```

*leo.collado@libd.org

```
##  package                * version  date       source
##  acepack                  1.4.1    2016-10-29 CRAN (R 3.4.0)
##  annotate                 1.55.0   2017-05-04 Bioconductor
##  AnnotationDbi          * 1.39.2   2017-07-26 Bioconductor
##  AnnotationFilter         1.1.3    2017-06-28 Bioconductor
##  AnnotationHub            2.9.5    2017-06-06 Bioconductor
##  assertthat               0.2.0    2017-04-11 cran (@0.2.0)
##  backports                1.1.0    2017-05-22 CRAN (R 3.4.0)
##  base                   * 3.4.1    2017-07-17 local
##  base64enc                0.1-3    2015-07-28 cran (@0.1-3)
##  bibtex                   0.4.2    2017-06-30 CRAN (R 3.4.1)
##  Biobase                * 2.37.2   2017-05-05 Bioconductor
##  BiocGenerics           * 0.23.0   2017-05-04 Bioconductor
##  BiocInstaller            1.27.2   2017-05-04 Bioconductor
##  BiocParallel             1.11.4   2017-06-27 Bioconductor
##  BiocStyle              * 2.5.8    2017-07-21 Bioconductor
##  BiocWorkflowTools        1.3.9    2017-06-09 Bioconductor
##  biomaRt                  2.33.3   2017-06-17 Bioconductor
##  Biostrings               2.45.3   2017-07-21 Bioconductor
##  biovizBase               1.25.1   2017-05-05 Bioconductor
##  bit                      1.1-12   2014-04-09 CRAN (R 3.4.0)
##  bit64                    0.9-7    2017-05-08 CRAN (R 3.4.0)
##  bitops                   1.0-6    2013-08-17 cran (@1.0-6)
##  blob                     1.1.0    2017-06-17 CRAN (R 3.4.0)
##  bookdown                 0.4      2017-05-20 CRAN (R 3.4.0)
##  BSgenome                 1.45.1   2017-05-05 Bioconductor
##  bumphunter             * 1.17.2   2017-05-20 Bioconductor
##  caTools                  1.17.1   2014-09-10 cran (@1.17.1)
##  checkmate                1.8.3    2017-07-03 CRAN (R 3.4.1)
##  cluster                  2.0.6    2017-03-10 CRAN (R 3.4.1)
##  clusterProfiler        * 3.5.5    2017-07-25 Bioconductor
##  codetools                0.2-15   2016-10-05 CRAN (R 3.4.1)
##  colorspace               1.3-2    2016-12-14 cran (@1.3-2)
##  compiler                 3.4.1    2017-07-17 local
##  curl                     2.8.1    2017-07-21 CRAN (R 3.4.1)
##  data.table               1.10.4   2017-02-01 cran (@1.10.4)
##  datasets               * 3.4.1    2017-07-17 local
##  DBI                      0.7      2017-06-18 CRAN (R 3.4.0)
##  DEFormats                1.5.0    2017-05-04 Bioconductor
##  DelayedArray           * 0.3.19   2017-07-28 Bioconductor
##  derfinder              * 1.11.5   2017-07-07 Bioconductor
##  derfinderHelper          1.11.0   2017-05-04 Bioconductor
##  derfinderPlot          * 1.11.0   2017-05-05 Bioconductor
##  DESeq2                 * 1.17.11  2017-07-22 Bioconductor
##  devtools               * 1.13.2   2017-06-02 CRAN (R 3.4.0)
##  dichromat                2.0-0    2013-01-24 cran (@2.0-0)
##  digest                   0.6.12   2017-01-27 CRAN (R 3.4.0)
##  DO.db                    2.9      2017-05-04 Bioconductor
##  doRNG                    1.6.6    2017-04-10 CRAN (R 3.4.0)
##  DOSE                   * 3.3.1    2017-06-02 Bioconductor
##  downloader               0.4      2015-07-09 CRAN (R 3.4.0)
##  DT                     * 0.2      2016-08-09 cran (@0.2)
##  edgeR                  * 3.19.3   2017-07-01 Bioconductor
##  ensembldb                2.1.10   2017-06-17 Bioconductor
##  evaluate                 0.10.1   2017-06-24 CRAN (R 3.4.1)
##  fastmatch                1.1-0    2017-01-28 CRAN (R 3.4.0)
##  fgsea                    1.3.1    2017-05-04 Bioconductor
##  foreach                * 1.4.3    2015-10-13 CRAN (R 3.4.0)
##  foreign                  0.8-69   2017-06-22 CRAN (R 3.4.1)
##  Formula                  1.2-2    2017-07-10 CRAN (R 3.4.1)
##  gdata                    2.18.0   2017-06-06 CRAN (R 3.4.0)
##  genefilter               1.59.0   2017-05-04 Bioconductor
##  geneplotter              1.55.0   2017-05-04 Bioconductor
##  GenomeInfoDb           * 1.13.4   2017-06-06 Bioconductor
##  GenomeInfoDbData         0.99.1   2017-07-17 Bioconductor
```

```
##  GenomicAlignments          1.13.4   2017-07-20 Bioconductor
##  GenomicFeatures          * 1.29.8   2017-07-20 Bioconductor
##  GenomicFiles               1.13.10  2017-07-17 Bioconductor
##  GenomicRanges            * 1.29.12  2017-07-28 Bioconductor
##  GEOquery                   2.43.0   2017-05-04 Bioconductor
##  GGally                     1.3.1    2017-06-08 CRAN (R 3.4.0)
##  ggbio                      1.25.3   2017-07-01 Bioconductor
##  ggplot2                  * 2.2.1    2016-12-30 CRAN (R 3.4.0)
##  git2r                      0.19.0   2017-07-19 CRAN (R 3.4.1)
##  GO.db                      3.4.1    2017-05-04 Bioconductor
##  GOSemSim                   2.3.1    2017-05-23 Bioconductor
##  gplots                   * 3.0.1    2016-03-30 cran (@3.0.1)
##  graph                      1.55.0   2017-05-04 Bioconductor
##  graphics                 * 3.4.1    2017-07-17 local
##  grDevices                * 3.4.1    2017-07-17 local
##  grid                       3.4.1    2017-07-17 local
##  gridExtra                  2.2.1    2016-02-29 CRAN (R 3.4.0)
##  gtable                     0.2.0    2016-02-26 CRAN (R 3.4.0)
##  gtools                     3.5.0    2015-05-29 cran (@3.5.0)
##  highr                      0.6      2016-05-09 cran (@0.6)
##  Hmisc                      4.0-3    2017-05-02 CRAN (R 3.4.0)
##  htmlTable                  1.9      2017-01-26 CRAN (R 3.4.0)
##  htmltools                  0.3.6    2017-04-28 CRAN (R 3.4.0)
##  htmlwidgets                0.9      2017-07-10 CRAN (R 3.4.1)
##  httpuv                     1.3.5    2017-07-04 CRAN (R 3.4.1)
##  httr                       1.2.1    2016-07-03 CRAN (R 3.4.0)
##  igraph                     1.1.2    2017-07-21 CRAN (R 3.4.1)
##  interactiveDisplayBase     1.15.0   2017-05-04 Bioconductor
##  IRanges                  * 2.11.12  2017-07-22 Bioconductor
##  iterators                * 1.0.8    2015-10-13 CRAN (R 3.4.0)
##  jsonlite                   1.5      2017-06-01 CRAN (R 3.4.0)
##  KernSmooth                 2.23-15  2015-06-29 CRAN (R 3.4.1)
##  knitcitations              1.0.8    2017-07-04 CRAN (R 3.4.1)
##  knitr                    * 1.16     2017-05-18 CRAN (R 3.4.0)
##  knitrBootstrap             1.0.1    2017-07-19 CRAN (R 3.4.1)
##  labeling                   0.3      2014-08-23 cran (@0.3)
##  lattice                    0.20-35  2017-03-25 CRAN (R 3.4.1)
##  latticeExtra               0.6-28   2016-02-09 CRAN (R 3.4.0)
##  lazyeval                   0.2.0    2016-06-12 cran (@0.2.0)
##  limma                    * 3.33.6   2017-07-26 Bioconductor
##  locfit                   * 1.5-9.1  2013-04-20 CRAN (R 3.4.0)
##  lubridate                  1.6.0    2016-09-13 CRAN (R 3.4.0)
##  magrittr                   1.5      2014-11-22 cran (@1.5)
##  markdown                   0.8      2017-04-20 cran (@0.8)
##  Matrix                     1.2-10   2017-05-03 CRAN (R 3.4.1)
##  matrixStats              * 0.52.2   2017-04-14 CRAN (R 3.4.0)
##  memoise                    1.1.0    2017-04-21 CRAN (R 3.4.0)
##  methods                  * 3.4.1    2017-07-17 local
##  mime                       0.5      2016-07-07 CRAN (R 3.4.0)
##  munsell                    0.4.3    2016-02-13 cran (@0.4.3)
##  nnet                       7.3-12   2016-02-02 CRAN (R 3.4.1)
##  org.Hs.eg.db             * 3.4.1    2017-05-04 Bioconductor
##  OrganismDbi                1.19.0   2017-05-05 Bioconductor
##  parallel                 * 3.4.1    2017-07-17 local
##  pheatmap                 * 1.0.8    2015-12-11 CRAN (R 3.4.0)
##  pkgconfig                  2.0.1    2017-03-21 CRAN (R 3.4.0)
##  pkgmaker                   0.22     2014-05-14 CRAN (R 3.4.0)
##  plyr                       1.8.4    2016-06-08 cran (@1.8.4)
##  prettyunits                1.0.2    2015-07-13 CRAN (R 3.4.0)
##  progress                   1.1.2    2016-12-14 CRAN (R 3.4.0)
##  ProtGenerics               1.9.0    2017-05-04 Bioconductor
##  qvalue                     2.9.0    2017-05-04 Bioconductor
##  R6                         2.2.2    2017-06-17 CRAN (R 3.4.0)
##  RBGL                       1.53.0   2017-05-04 Bioconductor
##  RColorBrewer             * 1.1-2    2014-12-07 cran (@1.1-2)
```

```
##  Rcpp                     0.12.12  2017-07-15 CRAN (R 3.4.1)
##  RCurl                    1.95-4.8 2016-03-01 cran (@1.95-4.)
##  recount                * 1.3.2    2017-07-29 Bioconductor
##  RefManageR               0.14.12  2017-07-04 CRAN (R 3.4.1)
##  regionReport           * 1.11.2   2017-07-15 Bioconductor
##  registry                 0.3      2015-07-08 CRAN (R 3.4.0)
##  rentrez                  1.1.0    2017-06-01 CRAN (R 3.4.0)
##  reshape                  0.8.6    2016-10-21 CRAN (R 3.4.0)
##  reshape2                 1.4.2    2016-10-22 cran (@1.4.2)
##  rlang                    0.1.1    2017-05-18 CRAN (R 3.4.0)
##  rmarkdown              * 1.6      2017-06-15 CRAN (R 3.4.0)
##  rngtools                 1.2.4    2014-03-06 CRAN (R 3.4.0)
##  rpart                    4.1-11   2017-03-13 CRAN (R 3.4.1)
##  rprojroot                1.2      2017-01-16 cran (@1.2)
##  Rsamtools                1.29.0   2017-05-05 Bioconductor
##  RSQLite                  2.0      2017-06-19 CRAN (R 3.4.1)
##  rstudioapi               0.6      2016-06-27 CRAN (R 3.4.0)
##  rtracklayer            * 1.37.3   2017-07-22 Bioconductor
##  rvcheck                  0.0.9    2017-07-10 CRAN (R 3.4.1)
##  S4Vectors              * 0.15.5   2017-06-27 Bioconductor
##  scales                   0.4.1    2016-11-09 cran (@0.4.1)
##  shiny                    1.0.3    2017-04-26 CRAN (R 3.4.0)
##  splines                  3.4.1    2017-07-17 local
##  stats                  * 3.4.1    2017-07-17 local
##  stats4                 * 3.4.1    2017-07-17 local
##  stringi                  1.1.5    2017-04-07 cran (@1.1.5)
##  stringr                  1.2.0    2017-02-18 cran (@1.2.0)
##  SummarizedExperiment   * 1.7.5    2017-06-22 Bioconductor
##  survival                 2.41-3   2017-04-04 CRAN (R 3.4.1)
##  tibble                   1.3.3    2017-05-28 CRAN (R 3.4.0)
##  tidyr                    0.6.3    2017-05-15 CRAN (R 3.4.0)
##  tools                    3.4.1    2017-07-17 local
##  utils                  * 3.4.1    2017-07-17 local
##  VariantAnnotation        1.23.6   2017-07-22 Bioconductor
##  withr                    2.0.0    2017-07-28 CRAN (R 3.4.1)
##  XML                      3.98-1.9 2017-06-19 CRAN (R 3.4.1)
##  xml2                     1.1.1    2017-01-24 cran (@1.1.1)
##  xtable                   1.8-2    2016-02-05 cran (@1.8-2)
##  XVector                  0.17.0   2017-05-04 Bioconductor
##  yaml                     2.1.14   2016-11-12 cran (@2.1.14)
##  zlibbioc                 1.23.0   2017-05-04 Bioconductor
```
