# Supplementary material for: recount workflow: Accessing over 70,000 human RNA-seq samples with Bioconductor
Source: F1000Res. 2017 Aug 24;6:1558. [Version 1] doi: 10.12688/f1000research.12223.1 (PMC5621122; doi:10.12688/f1000research.12223.1)
Supplement: Peer review history

# Peer review: Referee response for version 1

**DOI:** 10.5256/f1000research.13233.r25459

**Published:** 2017-09-27

**Authors:** Nicholas J. Schurch

**Affiliations:** 1 Division of Computational Biology, College of Life Sciences, University of Dundee, Dundee, UK

The authors present a workflow for working with the 70,000 processed RNA-seq datasets that form the recount2 project, using R, and seek to expand on the details presented in the original recount2 publication by describing how coverage count matrices were computed in recount2.

 I'm always slightly confused about the point of these workflow papers - this kind of example workflow information seems better suited to the R package vignettes, and for this reason I sometimes find them awkward to review. In addition, a considerably similar set of example workflow information (albeit somewhat less well described) has already been published in the supplementary information for the original recount2 publication from the same authors (doi:10.1038/nbt.3838, specifically see Supp. Text & Figures, and Supp. Notes 3 & 4) ^1^. Indeed, the supplementary info there goes further than this workflow in describing how to compare results from recount2 across several studies (Supp. Note 5). Personally I found the workflow example here somewhat convoluted and difficult to follow in places but I am sure the community will find it useful in helping to use the recount2 resource and perhaps the nature of such an example workflow presented on real data for a package such as this.

 Happily, however, the authors also present substantially new and more detailed information in a few key areas. In particular the description of how the recount2 read coverage matrices are computed is useful and interesting and the example showing how to supplement the project metadata with additional information is useful.

 Specific Comments:

 1. I don't really like the use of pseudo-maths equations for Eq1 & 2 - I'd like to see the words replaced with algebraic variables with meanings explained in the text.

 2. The scaled read counts are not the same as the actual mapping read counts that are typically required by downstream DE tools (which then typically apply their own appropriate normalization to these numbers). I'd like to see recount2 provide the actual mapping read count for features in addition to the scaled read counts. That said (and if I’m understanding things correctly) the manuscript here is a description of what the format of the recount2 data is and that has already been published - so I'm not expecting this to be changed.

 3. I really don't understand the sentence: "Not all differentially expressed genes have differentially expressed exons." Surely this is the definition of a DE gene?? I absolutely agree that "Moreover, genes with at least one differentially expressed exon are not necessarily differentially expressed" - differential transcript usage  is a prime example of where this can happen - but if a gene is DE I'm pretty sure that it must have a DE exon.

 4. I don't see the need for figs 28-36 - a single example of the plot type should be sufficient I think for an example workflow.

 5. It would be nice if recount2 could also provide information at the transcript level. Have the authors considered augmenting recount2 with salmon quantifications for all the data? (big job and more of a 'feature request' really).

I have read this submission. I believe that I have an appropriate level of expertise to confirm that it is of an acceptable scientific standard.

## References

[1] Collado-TorresLNelloreAKammersKEllisSETaubMAHansenKDJaffeAELangmeadBLeekJT: Reproducible RNA-seq analysis using recount2. *Nat Biotechnol*.2017;35(4) : 10.1038/nbt.3838 319-321 10.1038/nbt.3838 28398307PMC6742427

# Peer review: Referee response for version 1

**DOI:** 10.5256/f1000research.13233.r25458

**Published:** 2017-09-25

**Authors:** Alejandro Reyes

**Affiliations:** 1 Department of Biostatistics and Computational Biology, Dana-Farber Cancer Institute, Boston, MA, USA

The manuscript by Collado-Torres *et al*. provides a workflow to analyze public RNA-seq data using ‘recount2’. Recount2 is a resource that provides whole-genome coverage tracks for more than 70,000 RNA-seq experiments. The accompanying R/Bioconductor package ‘recount’ gives programmatic access to download read counts per gene and to estimate read counts for genomic regions of interest. In an RNA-seq pipeline, processing raw data into the formats available through recount2 involves the most time-consuming steps. Thus, recount2 will save many researchers a lot of time.

 The workflow describes how to programmatically access data from recount2 and describes different analyses that can be done using these data. However, I think the authors needs improve and clarify some aspects of the workflow, which I summarize below.

 Major comments:

  

1. The authors use the formula in equation 1 to scale read counts. While I agree that the read counts will be approximately equal to the sum of the coverage divided by the read length, it was not clear why the additional rescaling is needed. I recommend that the authors include a more extensive justification. Also, if the experiments were paired-end, wouldn’t this formula be counting reads instead of sequenced RNA fragments (i.e. double counting)?
2. The section “Enriching annotation” describes several functions and analyses but does not provide any code or examples. Currently, since it is incomplete, it is more distracting than informative. I suggest that the authors either expand this section and add code or drop it.
3. I don’t understand the biological question behind a differential expression analysis at the exon level. Could the authors clarify what the biological question is? If the aim is to find differential exon usage, wouldn’t it be better to use either DEXSeq, DRIMseq, or similar packages that are specifically designed for this analysis?

 

 Minor comments:

  

1. The first three sentences of the introduction need references.
2. The sentence “generally, when investigators use the term expression, they refer to gene expression” is not entirely true. For example, developmental or cell biologists often interpret “expression” as protein expression.
3. For full reproducibility, it would be useful to download the data within R using the SRAdb package instead of downloading it manually.
4. The code that creates the age groups is too complicated (4 embedded ‘ifelse’ statements). I have submitted a pull request with a simplified version of it ( https://github.com/LieberInstitute/recountWorkflow/pull/1).
5. Figures 13 and 14 could be merged into a single plot, using shapes and colors to distinguish the different annotations. The same holds for figures 23 and 24.

I have read this submission. I believe that I have an appropriate level of expertise to confirm that it is of an acceptable scientific standard, however I have significant reservations, as outlined above.

# Peer review: Referee response for version 1

**DOI:** 10.5256/f1000research.13233.r25456

**Published:** 2017-09-12

**Authors:** Davide Risso

**Affiliations:** 1 Division of Biostatistics and Epidemiology, Department of Healthcare Policy and Research, Weill Cornell Medicine, New York, NY, USA

The authors present a workflow that describes how to analyze the datasets available through the Recount2 project with Bioconductor. Since many of the state-of-the-art methods for the analysis of RNA-seq data are implemented in R and available through the Bioconductor project, this contribution is an important resource for researchers interested in reanalyzing the impressive amount of data that the authors have processed in the Recount2 project.

 I have a few comments that hopefully will help improve the workflow.

 1. I was a bit confused by the rationale of the scaled coverage counts. And especially on the need for a target library size and the use of scaled counts. Wouldn't it be simpler to divide the coverage by read length (without rescaling)? Wouldn't that result in the actual reads mapped to each region (exon, gene, ...)? I understand that for the derfinder analysis, some rescaling is needed for normalization purposes, but for more "classic" analysis (such as gene- or exon-level differential expression) where the counts are normalized later in the workflow, wouldn't starting from 'coverage/readlength' be a more sensible choice?

 2. Sex prediction. This is a really interesting part of the analysis, even though it's not the focus of this workflow. It would be interesting to get the authors' opinion of how to best use this feature on real analyses. For instance, are the 8 misclassified samples likely to be false positives from the classifiers or are they mislabeled samples? What is the recommendation of the authors in such cases? Should these samples be discarded or is there any diagnostics that can be run to make sure that the quality of these samples is not compromised?

 3. I think that the authors should give more details on the design matrix. For instance, why did they decide to include RIN and sex? Why is it important to include these variables in the model? More generally, the workflow lacks details on the limma pipeline. I understand that this is not the focus of the authors' work, but it may be confusing for beginners that don't have a direct experience with limma or voom. The authors could for instance refer the reader to the limma workflow ^1^ for details.

 4. Similarly, there is a lack of details on the GO enrichment analysis. Since there are many types of gene-set enrichment analysis, a paragraph could be added with more details and perhaps some references to explain what enrichment analysis is and what types of hypotheses are tested.

 5. One important advantage of exon-level differential expression is that it can be used to infer alternative splicing. This can be done with the functions 'diffSplice()' and 'topSplice()' in limma or with the DEXSeq package. It would be nice to showcase these functions or at least to mention that they exist.

 6. Is the annotation in Recount2 stable? Or is it constantly updated when a new version of Gencode is released? If the former, it might make sense to package the 'gencode_v25_hg38_txdb' object that the authors create in the workflow so that each user does not have to create it from scratch every time.

 Minor comments:

 - The authors use throughout the paper 'assays(rse)$counts' to access the counts of the 'rse' object. Although this is correct, a clearer and more concise way is 'assay(rse)' (or 'assay(rse, "counts")' if the authors want to explicitly state the name of the assay).

 - Section "Coverage counts provided by recount2". The authors say "Although recount can generate count matrices for other annota- tions using hg38 coordinates. " But they never say how this can be done. It would be good to add a paragraph on how to do that (which I presume involves creating an alternative txdb object).

I have read this submission. I believe that I have an appropriate level of expertise to confirm that it is of an acceptable scientific standard, however I have significant reservations, as outlined above.

## References

[1] LawCWAlhamdooshMSuSSmythGKRitchieME: RNA-seq analysis is easy as 1-2-3 with limma, Glimma and edgeR. *F1000Res*.2016;5: 10.12688/f1000research.9005.2 1408 10.12688/f1000research.9005.2 27441086PMC4937821
